# Delineation of a thrombin receptor-stimulated vascular smooth muscle cell transition generating cells in the plaque-stabilizing fibrous cap

**DOI:** 10.1093/cvr/cvaf112

**Published:** 2025-06-27

**Authors:** James C K Taylor, Matthew D Worssam, Sebnem Oc, Jordi Lambert, Krishnaa T Mahbubani, Kirsty Foote, Allie Finigan, Yee-Hung Chan, Nichola Figg, Murray C H Clarke, Martin R Bennett, Helle F Jørgensen

**Affiliations:** Section of Cardiorespiratory Medicine, Department of Medicine, University of Cambridge, Victor Phillip Dahdaleh Heart and Lung Research Institute, Papworth Road, Cambridge CB2 0BB, UK; Section of Cardiorespiratory Medicine, Department of Medicine, University of Cambridge, Victor Phillip Dahdaleh Heart and Lung Research Institute, Papworth Road, Cambridge CB2 0BB, UK; Section of Cardiorespiratory Medicine, Department of Medicine, University of Cambridge, Victor Phillip Dahdaleh Heart and Lung Research Institute, Papworth Road, Cambridge CB2 0BB, UK; Functional Gene Control Group, MRC Laboratory of Medical Sciences and Institute of Clinical Sciences, Faculty of Medicine, Imperial College Du Cane Road, London W12 0NN, UK; Section of Cardiorespiratory Medicine, Department of Medicine, University of Cambridge, Victor Phillip Dahdaleh Heart and Lung Research Institute, Papworth Road, Cambridge CB2 0BB, UK; Collaborative Biorepository for Translational Medicine, Department of Surgery, University of Cambridge and NIHR Cambridge Biomedical Research Centre, Cambridge CB2 0QQ, UK; Section of Cardiorespiratory Medicine, Department of Medicine, University of Cambridge, Victor Phillip Dahdaleh Heart and Lung Research Institute, Papworth Road, Cambridge CB2 0BB, UK; Section of Cardiorespiratory Medicine, Department of Medicine, University of Cambridge, Victor Phillip Dahdaleh Heart and Lung Research Institute, Papworth Road, Cambridge CB2 0BB, UK; Section of Cardiorespiratory Medicine, Department of Medicine, University of Cambridge, Victor Phillip Dahdaleh Heart and Lung Research Institute, Papworth Road, Cambridge CB2 0BB, UK; Section of Cardiorespiratory Medicine, Department of Medicine, University of Cambridge, Victor Phillip Dahdaleh Heart and Lung Research Institute, Papworth Road, Cambridge CB2 0BB, UK; Section of Cardiorespiratory Medicine, Department of Medicine, University of Cambridge, Victor Phillip Dahdaleh Heart and Lung Research Institute, Papworth Road, Cambridge CB2 0BB, UK; Section of Cardiorespiratory Medicine, Department of Medicine, University of Cambridge, Victor Phillip Dahdaleh Heart and Lung Research Institute, Papworth Road, Cambridge CB2 0BB, UK; Section of Cardiorespiratory Medicine, Department of Medicine, University of Cambridge, Victor Phillip Dahdaleh Heart and Lung Research Institute, Papworth Road, Cambridge CB2 0BB, UK

**Keywords:** Vascular smooth muscle cells, Atherosclerosis, Fibrous cap, Phenotypic switching, Protease-activated receptor-1

## Abstract

**Aims:**

Vascular smooth muscle cells (VSMCs) accumulate in atherosclerotic plaques and exhibit remarkable phenotypic plasticity, contributing to both plaque growth and stability. The plaque-stabilizing fibrous cap is rich in VSMC-derived cells, yet the cellular transitions and regulatory mechanisms governing fibrous cap formation remain unclear. Here, we aimed to identify the VSMC phenotypic transitions associated with this critical process.

**Methods and results:**

Mapping of lineage-traced VSMCs during plaque development revealed investment of VSMCs prior to fibrous cap formation. Using single-cell RNA-sequencing (scRNA-seq) profiles of lineage-traced VSMCs from atherosclerotic and acutely injured mouse arteries, we identified a disease-specific VSMC state co-expressing contractile genes with extracellular matrix (ECM) components (including fibrillar collagens and elastin) and NOTCH3, which are associated with fibrous cap formation. Computational trajectory analysis predicted that this proposed fibrous cap-related VSMC (fcVSMC) state arises from a previously described plastic, intermediate VSMC population expressing SCA1 and VCAM1. Clonal analysis further showed that NOTCH3^+^ fcVSMCs derive from intermediate VSMCs in both atherosclerosis and an acute vascular injury model, suggesting a conserved disease-relevant mechanism. The fcVSMCs were enriched in plaque fibrous caps compared to lesion cores, consistent with a role in fibrous cap formation. By combining scRNA-seq trajectory analysis and spatial transcriptomics of human atherosclerotic plaques, we identified protease-activated receptor-1 (PAR1) as a candidate regulator of fcVSMC generation. PAR1 was expressed by VSMCs in human plaque fibrous caps and PAR1 activation by thrombin induced expression of contractile genes and ECM components associated with the fcVSMC state in human VSMCs.

**Conclusion:**

Our findings identify a VSMC transition linked to fibrous cap formation in atherosclerosis and show this is modelled by vascular injury. We identify VSMC-expressed PAR1 as a potential therapeutic target for promoting plaque stability by driving the transition to the matrix-producing, fibrous cap-associated VSMC state.

## Graphical Abstract

**Graphical Abstract:**
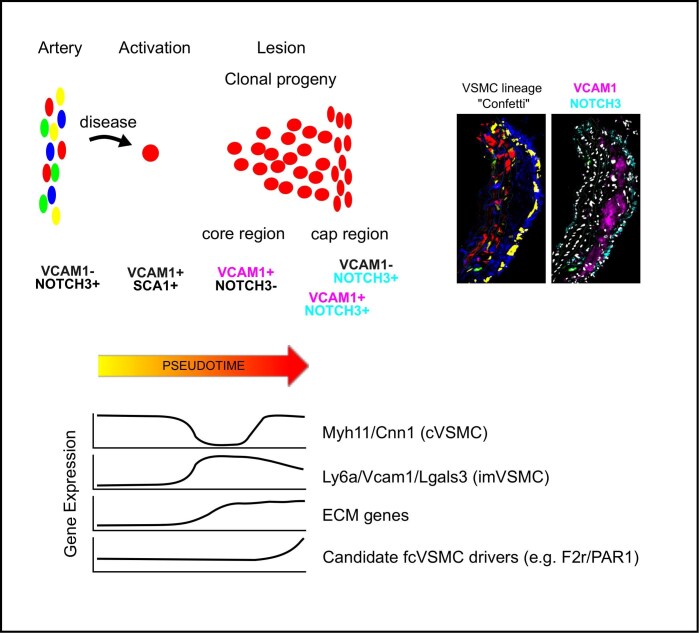

**Time of primary review: 72 days**

## 1. Introduction

Vascular smooth muscle cell (VSMC) accumulation is a hallmark of atherosclerosis^1,2^ and neointima formation following vessel occlusion and injury.^3,4^ VSMCs are quiescent and express components of the contractile machinery (such as *MYH11*, *CNN1*, and *ACTA2*) to control blood pressure and flow in healthy arteries.^1^ However, these cells retain remarkable plasticity and dedifferentiate in response to injury or inflammation. This involves down-regulating the contractile apparatus and increasing migration, proliferation, and extracellular matrix (ECM) production. In atherosclerotic plaques, VSMCs acquire a range of different phenotypes^1,5,6^ and generate both the plaque-stabilizing fibrous cap and cells in the lesion core that have characteristics of mesenchymal cells, phagocytes, and osteochondrocytes, which may contribute to lesion destabilization.^2,6^ Delineating the governance of these distinct VSMC-derived cells in disease will therefore aid understanding of the functional role of these cells in disease.

VSMC-contribution to atherosclerotic plaques and injury-induced neointimal lesions in disease models is oligoclonal.^7–10^ Very few pre-existing VSMCs give rise to all lesional VSMCs, and the range of plaque VSMC phenotypes can be generate from the clonal progeny of individual cells.^7^^,11^ In mice, VSMCs with reduced levels of contractile markers that express stem cell antigen 1 (SCA1, encoded by *Ly6a*) and Vcam1 have been suggested to represent a plastic VSMC state capable of giving rise to diverse plaque VSMC phenotypes.^11,12^ Humans, which do not have a SCA1/*Ly6a* homologue, also have VSMC populations with an equivalent transcriptional profile to mouse SCA1+ VSMCs.^13^ This plastic VSMC state is rarely detected in healthy arteries and is induced upon VSMC de-differentiation in response to disease stimuli.^11,13^ In atherosclerosis, such cells showed substantial overlap in the transcriptional signature of ‘activated’ VSMC-derived cells expressing osteochondrocyte and phagocyte markers.^11^ Computational inference of VSMC transdifferentiation pathways, functional *in vitro* experiments with isolated SCA1-expressing VSMCs and use of a dual lineage tracing system have all provided substantial evidence that VSMCs co-expressing SCA1, VCAM1, and LGALS3 act as intermediates and give rise to the diverse VSMC states observed in atherosclerotic plaques.^12,14,15^ Accordingly, this ‘intermediate modulated’ VSMC (imVSMC) population could represent a novel therapeutic target, whereby its disease-induced transdifferentiation to diverse cell states could be biased towards those states which are beneficial to disease outcome.

The fibrous cap structure is imperative to mitigating plaque rupture and downstream cardiovascular events, and is rich in VSMCs expressing typical contractile VSMC markers, as well as ECM components such as fibrillar collagens and elastin.^1,16,17^ Previous work has highlighted several candidate factors involved in promoting the formation of a VSMC-rich fibrous cap, such as PDGFRB,^18^ TCF21,^19^ and retinoic acid.^12^ Compelling evidence suggests that although loss of NOTCH3 signalling characterises VSMC lesion investment, subsequent reactivation is critical in the formation of a fibrous cap with contractile marker-expressing VSMCs.^20^ However, the VSMC states underpinning fibrous cap formation and how cap formation is related to the imVSMC state remains to be elucidated.

Here, we investigated the regulation of VSMCs underlying fibrous cap formation in atherosclerosis. By combining scRNA-seq analysis, and immunostaining in disease models with clonal VSMC lineage tracing we find a transcriptional VSMC state with characteristics of fibrous cap cells and show evidence that this is generated from the intermediate VSMC state. Interestingly, this transition was detected in both atherosclerotic and acutely injured arteries, suggesting importance for general VSMC regulation. We identify candidate regulators of this fibrous cap-associated transition and highlight VSMC-expressed thrombin receptor as a candidate driver of fibrous cap formation.

## 2. Methods

Experimental details are provided as Supplementary Information.

### 2.1 Animals and procedures

Experiments, licenced under UK Home Office regulations (P452C9546 and PP7513347), were approved by the University of Cambridge Ethical Reviews Committee. Tamoxifen-induced VSMC lineage-labelling of Myh11-CreER^T2^; Rosa26-Confetti (Myh11^Confetti^) and Myh11-CreER^T2^; Rosa26-eYFP (Myh11^eYFP^) animals was done before disease induction. Animals received pre-operative analgesic (∼0.1 mg/kg body weight, buprenorphine), were anaesthetized with 2.5%–3% isoflurane by inhalation (1.5 L/min) and the left carotid artery ligated with a 6–0 silk suture. Myh11^Confetti^; *Apoe^−/−^* mice were fed a high fat diet (HFD, Special Diets Services; 21% fat, 0.2% cholesterol). Animals were euthanized by cervical dislocation or CO_2_ asphyxiation.

### 2.2 Human tissue

Human tissue was acquired under informed consent using protocols approved by the Cambridge or Huntingdon Research Ethical Committee, conforming to the principles of the Declaration of Helsinki. Anonymized samples were obtained from patients undergoing carotid endarterectomy, cardiac transplant, valve replacement or by donation after circulatory death. Human arterial sections were co-stained for αSMA (DAKO, M0851, 1:400) and PAR1 (Novus Biologicals, N2–11, 1:200). A plaque-containing aortic sample from a 54-year old male organ donor was analysed by spatial transcriptomics (see Supplementary Information).

### 2.3 Arterial VSMC analysis

Plaque VSMC infiltration was analysed using 100 μm sections from the ascending and descending aorta, aortic arch and carotid arteries of 7 animals (4 at week 6.5 and 3 at week 11), previously used to assess VSMC clonality.^13^ A total of 150 unique plaques were analysed (see Supplementary material online, *Table S1*, plaques with suboptimal clearing were excluded). Lesion classification was based on DAPI signals, and Confetti signals subsequently used to score VSMC location, number, and clonality. Thin (14 μm) sections were stained with antibodies to NOTCH3 (Abcam, ab23426, 10 μg/mL), VCAM1 (BioLegend, 105702, 10 μg/mL) or isotype control antibodies, DAPI-stained (1 μg/mL), and mounted in RapiClear 1.52. VCAM1 and/or NOTCH3 expression by individual Confetti+ VSMCs was assessed by *Z-*plane navigation, and scored for 16 plaques (26 clones) after 11 weeks HFD (*n* = 3, Supplementary material online, *Table S1*) and vessels from control or injured animals (three per time point).

### 2.4 Cell culture and molecular analysis

Human VSMCs (hVSMCs), cultured in SMC-GM2 medium (Promocell) supplemented with 100 U/mL penicillin, 100 µg/mL streptomycin, were analysed at passages 2–10 and treated with thrombin (0.5, 1, or 2 U/mL) and Factor Xa (2.5 μg/mL) in serum-free media. Cells were transfected with 50 nM human PAR1-specific siRNA (ON-TARGETplus® SMART Pool, Dharmacon, L-005094–00-0005) or non-targeting control siRNA (Dharmacon, D-001810-10-05) for 72 h, before thrombin treatment. Western blotting was done using primary antibodies for PAR1 (Santa Cruz, ATAP2, 1:2000), and GAPDH (Cell Signaling Technologies, 2118, 1:4000). Primer sequences for reverse transcription, quantitative PCR (RT-qPCR) are listed in Supplementary material online, *Table S2*.

Bulk RNA-seq was conducted using female hVSMCs from three different donors, following 24-h thrombin/vehicle treatment in serum-free medium and data analysed using the limma package^21^ with cell line donor as a covariate.

ScRNA-seq profiles of VSMCs from mouse carotid arteries were generated as described^13^ after pooling single cell suspensions from 5 Myh11^eYFP^ animals 11 days post-ligation or 2 control Myh11^eYFP^ animals that did not undergo surgery. Gene count matrices for lineage-labelled VSMCs from a mouse atherosclerosis model^12^ were downloaded (GSE155513). Parameters for data processing and filtering are described in Supplementary material online, *Table S3*. Normalized, clustered and differential gene expression analysis was done in Seurat.^22^ Trajectory analysis was done using the Slingshot,^23^ Monocle3, or Partition-based graph abstraction^24^ in Scanpy^25^ with R-Python interfacing. Differential expression across pseudotime was performed with generalized additive modelling (GAM). Genes showing statistically significant pseudotime-dependent changes (logFC > 0.25) were clustered by Pearson correlation distance to reveal similar expression patterns. NicheNet (v2.2.0) was used in the sender-agnostic mode to identify potential ligands inducing the I-cluster 6 VSMC state.

Gene ontology (GO) analysis of genes differentially expressed between clusters, conditions, and cell states, or along trajectory path pseudotimes was carried out using gProfiler (https://biit.cs.ut.ee/gprofiler/gost) and the combined GO analysis was performed using the compareCluster function in clusterProfiler.^26^ Statistical significance was evaluated using cumulative hypergeometric probability testing with g:SCS multiple testing correction and 0.05 *P*-adj threshold. Circos plots were generated using the GOplot package (CRAN).

### 2.5 Statistical analysis

Statistical significance was assessed using Chi-squared (X^2^), Kruskal–Wallis (and Mann–Whitney between groups, with Bonferroni-correction, if *P* < 0.05), and cumulative hypergeometric probability testing, with g:SCS correction. Group sizes represent biologically independent samples (different donors, different mice, plaques or clones as specified), rather than technical replicates. An adjusted *P*-value (*P*-adj) <0.05 was considered statistically significant. Statistical testing information for single-cell and bulk RNA-seq dataset analysis is above and detailed in the Supplemental Information.

## 3. Results

### 3.1 Plaque VSMC infiltration leading to generation of the fibrous cap

**Figure 1:**
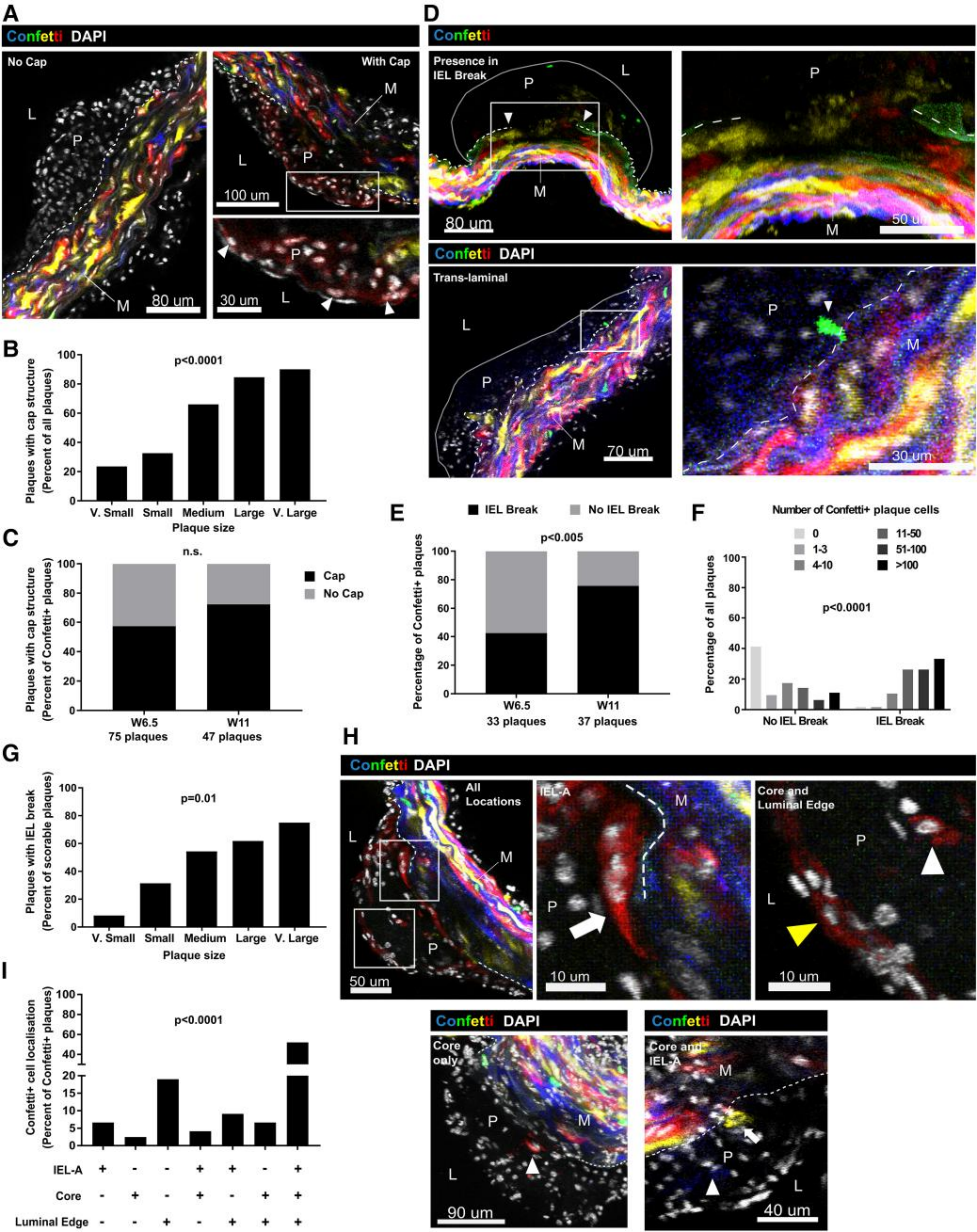
VSMC infiltration in atherosclerosis occurs prior to generation of a fibrous cap. Analysis of Confetti + cells in plaques from VSMC-lineage-labelled Myh11^Confetti^; *Apoe^−/−^* animals fed a HFD for 6.5 or 11 weeks (*W*). (*A*) Representative confocal images (single *Z*-stack) of plaques without (left) and with fibrous cap structure (arrowheads, right). Magnified view of the boxed region (lower right). Scale bars = 80 μm (left), 100 μm (top right), and 30 μm (lower right). (*B*) Percent of all plaques with fibrous cap structure according to plaque size. *n* = 150 plaques; *n* = 17 very (v) small, 49 small, 47 medium, 27 large, 10 v. large. *P* < 0.001, X^2^, and df = 4. (*C*) Percent of plaques containing Confetti^+^ cells stratified by time of HFD feeding. *n* = 122 plaques; *n* = 75 plaques from four animals at W6.5 and *n* = 47 plaques from three animals at W11. *P* = 0.09, X^2^, and df = 1. (*D*) Representative plaques (max. projection) with Confetti^+^ cells at an IEL break (top) or ‘translaminal’ Confetti^+^ cells crossing an intact IEL (lower panels). Magnified views show boxed regions (right). Arrowheads: IEL breakpoints (top) and translaminal Confetti^+^ cell (lower). Scale bars = 80 μm (top-left), 50 μm (top-right), 70 μm (bottom-left), and 30 μm (bottom-right). (*E*) Proportion of Confetti^+^ cell-containing plaques with an IEL break, stratified by time of HFD. *n* = 70 plaques from four animals (W6.5) and 37 plaques from three animals (W11). *P* = 0.005, X^2^, and df = 1. (*F*) Distribution of plaques by number of Confetti^+^ cells per plaque, for lesions without (54 plaques) or with an IEL break (43 plaques). *P* < 0.0001, X^2^, and df = 5. (*G*) Percent of plaques with IEL break, stratified by plaque size (9 v. small, 29 small, 35 medium, 17 large, 7 v. large). *P* = 0.04, X^2^, and df = 4. (*F*, *G*) Plaques were excluded if IEL status could not be confidently scored. (*H*) Representative confocal images (top, single Z-plane; lower panels, max. projection) of plaques showing Confetti^+^ cells in all plaque regions (top), core only (lower left), or core and ‘IEL-adjacent’ (IEL-A, lower right). Magnified views of boxed regions show IEL-A (middle) and ‘core and luminal edge’ located Confetti^+^ cells (right). Confetti^+^ cells at the IEL-A (white arrows), Confetti^+^ cells at the luminal edge (yellow arrowhead), and Confetti^+^ cells in the plaque core (white arrowheads) are marked. (*I*) Distribution of plaque regions with Confetti^+^ cells for plaques with Confetti^+^ cell investment (*n* = 122 plaques). *P* < 0.0001, X^2^, and df = 6. (*A*, *D*, *H*) Confetti (CFP = blue, RFP = red, YFP = yellow, and GFP = green) and DAPI (white) signals are shown as indicated. Plaque outlines (grey line) and internal elastic lamina (IEL, dashed line) are marked. L, lumen; P, plaque, M, media.

To understand the kinetics of fibrous cap formation, we analysed developing plaques in arteries of *Apoe^−/−^* mice that were fed a HFD for 6.5 (W6.5) or 11 weeks (W11). To trace the clonal progeny of VSMCs in lesions, we combined a Myh11-driven, tamoxifen-inducible Cre recombinase with the Rosa26-Confetti reporter (Myh11^Confetti^), and induced stochastic expression of one of four fluorescent proteins specifically in VSMCs prior to HFD feeding. Confocal microscopy of thick (100 μm) arterial sections revealed that the proportion of lesions with longitudinally arranged nuclei adjacent to the endothelium at the luminal edge, indicating formation of a fibrous cap, increased with both time of HFD and plaque size (*Figure 1A–C*). This is consistent with fibrous cap development correlating with the duration of lipid exposure and plaque volume.^16,27^ We focused on plaques that contained lineage-labelled VSMCs (122/150). A cap was observed in most, but a substantial percentage of lesions containing Confetti^+^ cells (36%) did not have an obvious cap structure. Plaques lacking a fibrous cap were observed more frequently at the earlier time point (42% at W6.5 *vs.* 28% at W11, *Figure 1C*), indicating that VSMCs can enter the plaque prior to fibrous cap formation.

Definitive scoring of internal elastic lamina (IEL) integrity was possible in 70 plaques with Confetti + cells, and an IEL break was observed in most of these (60%, *Figure 1D* and *E*). However, no IEL breaks were detected in 40% of lesions, and an intact IEL was most frequently observed in plaques at W6.5 (60%; *Figure 1E*). In two cases, we detected a translaminal lineage-labelled cell in lesions without IEL breaks (*Figure 1D*). This was a relatively rare event, but agree with migration of VSMCs through IEL fenestrations observed by high-resolution confocal microscopy.^28^ These findings suggest that VSMC plaque infiltration can occur both via cell migration through an intact IEL from the underlying media and through IEL breaks. Plaques without detectable IEL breaks tended to contain lower numbers of VSMC-lineage cells, whereas lesions with IEL breaks contained more VSMC lineage cells (*Figure 1F*). This suggests that VSMC investment through breaks in the IEL occurs in more advanced plaques, or that IEL breaks are secondary to VSMC investment. The frequency of IEL breaks for all (Confetti^+^ and Confetti^−^) plaques was higher at W11 (29/42, 69%) compared to W6.5 (14/52, 27%) and we observed a plaque size-dependent increase in IEL-break frequency (*Figure 1G*), further suggesting that IEL breaks are associated with plaque maturity.

To understand the dynamics of VSMC localization within plaques during lesion development, we scored the presence of Confetti^+^ cells in each of three plaque regions: ‘IEL-adjacent’ (IEL-A: plaque core, directly contacting the IEL), non-attached cells in the ‘core’ and ‘luminal edge’, which include the fibrous cap (*Figure 1H*). Plaques with VSMCs in all three locations were most common (63 out of 121 scorable Confetti^+^ lesions), although VSMC investment was limited to specific regions in 48% the plaques (*Figure 1I*). VSMCs were selectively located at the luminal edge in 23 plaques (19%, *Figure 1I*), which is in keeping with a previous study that suggested VSMCs initially migrate through the lesion via the luminal edge.^10^ However, lineage-labelled VSMC contribution at the luminal edge was absent in 16 lesions (13%), including 5 lesions with labelled cells selectively in the region adjacent to the IEL (IEL-A, *Figure 1I*). Overall, these findings suggest that, while migration via the luminal edge is prevalent, alternative routes exist and demonstrates that VSMCs are present in plaques before cap formation.

### 3.2 Inference of a VSMC transition that gives rise to a fibrous cap-associated cell state in vascular disease models.

**Figure 2:**
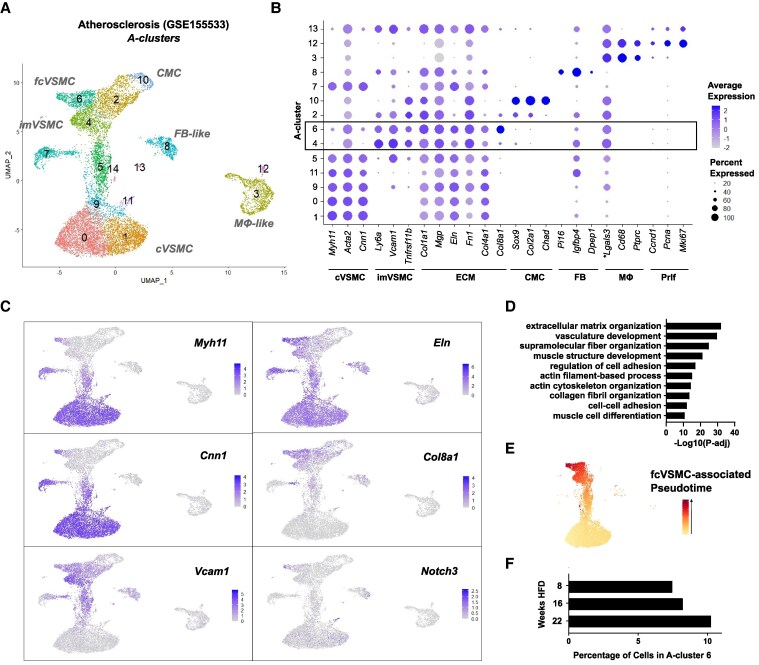
Identification of a proposed fibrous cap-associated VSMC state derived from intermediate modulated VSMCs. (*A*) Uniform manifold approximation and projection (UMAP) showing scRNA-Seq profiles of VSMC-lineage label-positive cells from atherosclerotic arteries of Myh11-CreER^t2^; reporter-ZsGreen; *Apoe^−/−^* animals (GSE155513)^20^ and healthy control Apoe^+/+^ carotid arteries. Atherosclerosis cell clusters (A-clusters) and VSMC states are annotated. (*B*) Dot plot showing expression of VSMC-state marker genes in clusters using a grey (low) to blue (high) scale and the percentage of cells with detected marker expression for each cluster indicated by dot size. cVSMC, contractile VSMC; imVSMC, intermediate modulated VSMC; ECM, extracellular matrix; CMC, chondromyocyte; FB, fibroblast; MΦ, macrophage; Prlf, proliferation. *Lgals3 is also expressed by imVSMCs. (*C*) UMAP feature plots showing expression levels for cVSMC (*Myh11, Cnn1),* ECM *(Eln, Col8a1, Col4a1)*, the imVSMC gene *Vcam1*, and *Notch3* using grey (low) to blue (high) scales. (*D*) GO terms enriched for genes up-regulated in A-cluster 6 compared to A-cluster 4 cells. *P*-adj: cumulative hypergeometric probability testing with g:SCS correction. (*E*) UMAP showing the cells included in the inferred trajectory generating A-cluster 6 cells. Pseudotime is indicated using a yellow–red scale. (*F*) Proportion of VSMC-lineage cells mapping to A-cluster 6 at each HFD time point.

While the VSMC origin of fibrous cap cells is well documented, the pathways underlying their generation are not fully understood. To capture active VSMC transitions underlying fibrous cap development, we therefore examined scRNA-seq profiles of lineage-traced VSMCs from experimental atherosclerosis at early-, mid- and late-stage (8, 16, and 22 weeks of HFD on *Apoe^−/−^* background; GSE155513^12^). Disease-specific VSMC states were identified by integration with profiles of VSMCs from healthy vessels of control Apoe^+/+^ mice. Contractile VSMC (cVSMC) markers (*Myh11, Acta2*, and *Cnn1*) were highly expressed in cell clusters found in both healthy and atherosclerotic arteries [Athero (A)-cluster 0, 1, 7, 9, and 11, *Figure 2A–C*; Supplementary material online, *Figure S1* and *Table S4*]. Disease-associated states^14,29^ included *Sox9^+^*, *Col2a1^+^*, and *Chad^+^* chondromyocytes (CMCs; A-cluster 2 and 10), fibroblast-like (FB-like, A-cluster 8) cells expressing high levels of *Pi16*, *Igfbp4*, and *Dpep1* and a distinct cell population expressing *Cd68* and other macrophage markers (MΦ-like; A-cluster 3 and 12; *Figure 2A* and *B*). We also detected activated, or intermediate modulated VSMCs (imVSMCs), characterized by expression of *Ly6a, Vcam1, Tnfrsf11b, Lgals3*, and ECM genes (A-cluster 4 and 5; *Figure 2B*, also been termed ‘SEM’,^12,14^ fibromyocytes,^19^ or pioneer cells^15^). A small population of proliferating (Prlf) VSMCs (*Ccnd1*^+^, *Pcna^+^*, and *Mki67^+^*) co-expressed imVSMC markers (A-cluster 13, *Figure 2A* and *B*), in keeping with previous work suggesting that the imVSMC state is associated with proliferation.^12^

An unannotated disease-specific cell cluster (A-cluster 6) co-expressed contractile VSMC markers (*Myh11, Acta2,* and *Cnn1*) with several atherosclerosis-induced ECM genes (*Col1a1, Mgp, Eln, Fn1, Col4a1,* and *Col8a1*) and had reduced levels of *Ly6a, Vcam1*, and *Tnfrsf11b* compared to imVSMCs (*Figure 2A–C*; Supplementary material online, *Figure S1*). Genes that were up-regulated in A-cluster 6 cells compared to A-cluster 4/imVSMCs included *Notch3* that has been shown to play an important role in fibrous cap formation,^20^ and were enriched for GO terms associated with contractile/muscle function (muscle structure development, actin cytoskeleton organization, and muscle cell differentiation), and genes associated with ECM production (ECM organization, collagen fibril organization; *Figure 2C* and *D*; Supplementary material online, *Table S5* and *S6*). The co-expression of the broader contractile gene programme with a synthetic phenotype led us to hypothesize that A-cluster 6 cells were associated with the fibrous cap (referred to as fcVSMCs). Independent trajectory inference analyses suggested that imVSMCs are intermediates for fcVSMC formation (*Figure 2E*; Supplementary material online, *Figure S1C*). Furthermore, A-cluster 6 cells were present at a greater proportion at the later time point of HFD feeding (22 weeks; *Figure 2F*; Supplementary material online, *Figure S1*), aligning with fibrous cap formation occurring at later disease stages^16,27^ (*Figure 1B* and *C*). This is consistent with the idea that imVSMCs represent dedifferentiated VSMCs (or fibromyocytes) that migrate into lesions and give rise to the other VSMC-lineage plaque cells.

**Figure 3:**
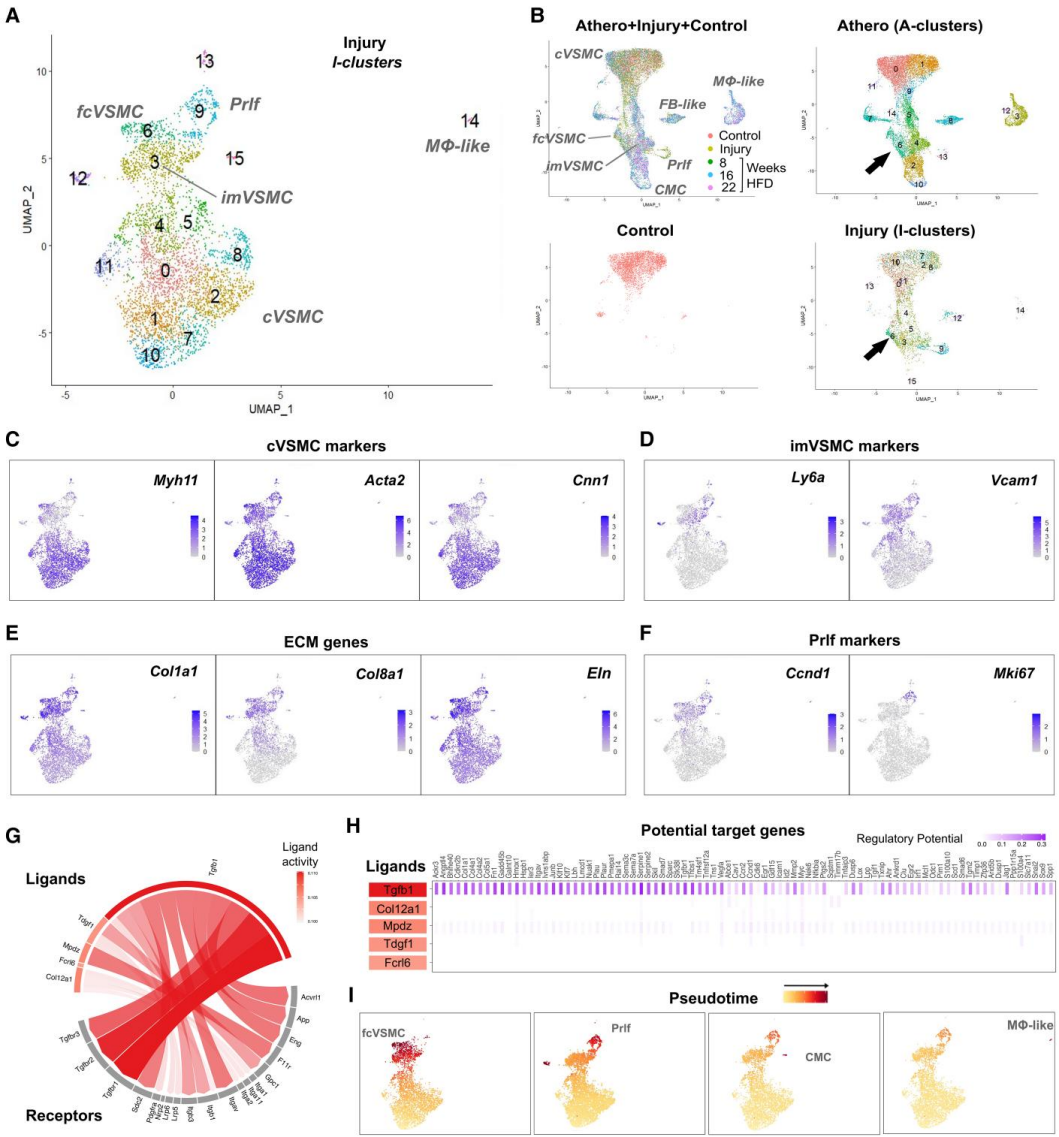
VSMCs generate the transcriptional fibrous cap-associated VSMC state 11 days after vascular injury. (*A*) Uniform manifold approximation and projection (UMAP) showing injury (I) cell cluster annotation and VSMC states for scRNA-Seq analysis of VSMC-lineage label positive cells from ligated left carotid arteries of Myh11^eYFP^ animals 11 days post-injury (DPI). (*B*) Integration of injury, atherosclerosis, and healthy control carotid arteries scRNA-Seq datasets. Top left: all cells coloured by dataset identity; top right: cells from the atherosclerosis data only, A-clusters annotated (see *Figure 2A*); lower left: cells from healthy arteries only, lower right: injury data, I-clusters annotated (panel A). (*C-F*) UMAP feature plots showing expression levels for markers of cVSMC (*C*), imVSMC (*D*), ECM (*E*), and proliferation *(F*) using grey (low) to blue scales (high). (*G, H*) Top 5 ligands and their associated receptors (*G*, arrow width indicates predicted strength) or regulatory potential for top 100 gene targets (*H*). Predicted ligand strength is indicated by shades of orange/red, regulatory potential in shades of purple. (*I*) UMAP showing the cells included the inferred trajectories leading to generation of a Prlf (I-cluster 9), CMC (I-cluster 15), MΦ-like or fibrous cap-associated (fcVSMC; I-cluster 6) cell state. Pseudotime is indicated using a yellow–red scale. cVSMC, contractile VSMC; imVSMC, intermediate modulated VSMC; CMC, chondromyocyte; FB, fibroblast; MΦ, macrophage; Prlf, proliferation.

We next compared the imVSMCs-to-fcVSMC transition to ‘re-differentiation’ of VSMCs after injury, using carotid ligation surgery, which results in reproducible and acute VSMC de-differentiation, formation of a VSMC-rich intimal lesion and subsequent upregulation of contractile VSMC markers.^30,31^ We performed scRNA-seq on VSMC lineage^+^ (eYFP^+^) cells sorted from ligated arteries of Myh11-CreERt2/Rosa26-eYFP (Myh11^eYFP^) mice 11 days post-injury (DPI), when neointimal lesions are developing and VSMC re-differentiation is ongoing^30,31^ (*Figure 3A*; Supplementary material online, *Figure S2*).

Interestingly, integration of the 11 DPI transcriptional profiles with atherosclerosis data (*Figure 3B*; Supplementary material online, *Figure S3*), suggested that the range of VSMC states induced by carotid ligation injury at this time point overlaps those observed in atherosclerosis. As shown in *Figure 2A* and *C-F*, injured arteries contain imVSMCs expressing *Ly6a^+^*, *Vcam1^+^*, and *Lgals3^+^* [injury cell cluster (I-cluster) 3 and 4] and Prlf cells expressing *Ccnd1^+^*, *Pcna^+^*, and *Mki67^+^* (I-cluster 9), in addition to cVSMCs with no, or low, response to injury (I-cluster 0, 1, 2, 7, 10, and 11), as expected.^13,32^ Interestingly, we also detected cells with high levels of *Sox9, Col2a1*, and *Chad*, that were similar to CMCs (I-cluster 15, Supplementary material online, *Figure S3*). Rare cells expressing FB-like (I-cluster 12, *Pi16^+^, Igfbp4^+^*, and *Dpep1^+^*) or MΦ-like genes (I-cluster 14, *Lgals3^+^, Cd68^+^*, and *Ptprc^+^*) had low expression of the *eYFP* lineage label (see Supplementary material online, *Figure S3*), suggesting that these cells were not derived from VSMCs, similar to what has been proposed in atherosclerosis.^29^

Additionally, a cell population co-expressing contractile genes with key ECM genes (*Col1a1, Mgp, Eln, Fn1, Col4a1*, and *Col8a1*) and *Notch3*, mapped together with the fcVSMC population from the atherosclerosis dataset (I-cluster 6; *Figure 3A* and *B*; Supplementary material online, *Figure S3*). This population was also present in an independent experiment at 11 DPI (see Supplementary material online, *Figure S4*), but was not detected at earlier timepoints (5 and 7 DPI),^13^ suggesting that these cells are derived from modulated cells and represent VSMC re-differentiation. Intercellular communication analysis predicted that TGF-beta, an important determinant of fibrous cap formation, is the top ligand for I-cluster 6 cells and that it has high regulatory potential for I-cluster 6 marker genes (*Figure 3G* and *H*). Computational trajectory analysis indicated that, similar to in atherosclerosis,^12,14^ imVSMCs act as an intermediate state after injury that generate the fcVSMCs as well as proliferating cells and other VSMC-derived subsets (*Figure 3I*; Supplementary material online, *Figure S2B* and *C*). Together, these data support the idea that VSMCs transition to disease-relevant states via an imVSMC state and suggest that these transitions can be investigated using the reproducible vascular injury model. Importantly, our analysis identified a novel imVSMC-derived fcVSMC state in both models, that we hypothesise is implicated in fibrous cap formation.

### 3.3 VSMC transition to a NOTCH3^+^ fibrous cap-associated cell state occurs in the fibrous cap and injury-induced lesions

**Figure 4:**
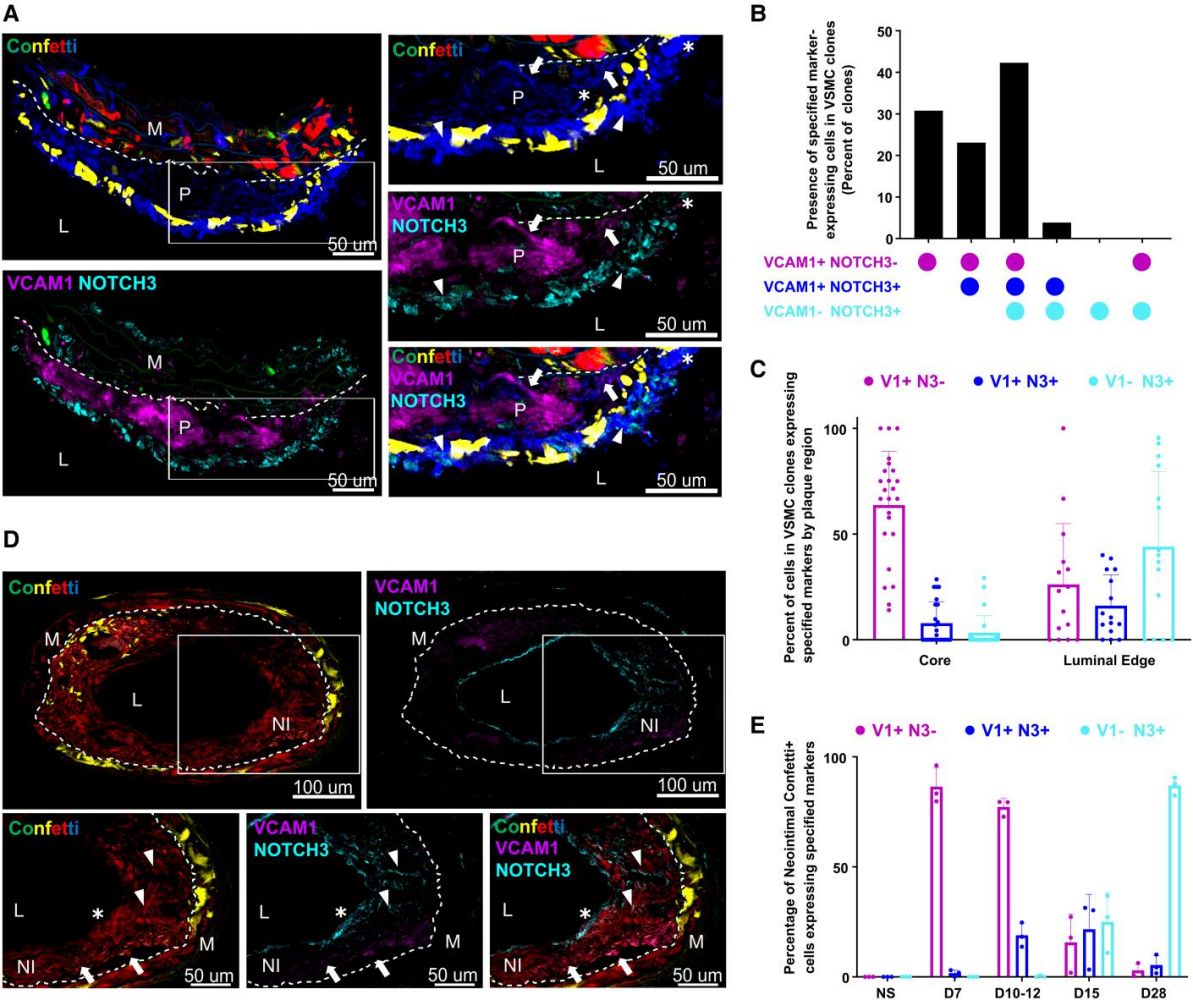
VSMCs in the intermediate modulated and fibrous cap-associated state co-occur in clonally related cells within lesions. (*A-C*) Immunofluorescence staining for VCAM1 and NOTCH3 in cryosections of a plaque from VSMC-lineage-labelled Myh11^Confetti^; *Apoe^−/−^* animals. (*A*) Representative image of plaque, showing the entire plaque (left) or magnified views of boxed region (right). Examples of VCAM1^+^ (arrows), NOTCH3^+^ (arrowheads), and VCAM1^+^NOTCH3^+^ cells (asterisk) are indicated. Scale bars = 50 µm. (*B*) Percentage of plaque-located Confetti^+^ clones that contain cells expressing specified combinations of markers. Dots represent clones (*n* = 26) from 16 plaques in three animals. (*C*) Percentage of cells with specified combinations of marker expression quantified separately for either the core or luminal edge portion of individual Confetti^+^ clones. *n* = 23 (core region) and 15 clones (luminal edge). Bars show mean, error-bars SD. (*D, E*) Immunofluorescence staining for VCAM1 and NOTCH3 in cryosections of the ligated artery of VSMC-lineage-labelled Myh11^Confetti^ animals. (*D*) Representative image (15 days post-injury) showing entire section (top; scale bar = 100 µm) and magnified views of boxed regions (bottom; scale bar = 50 µm). Examples of VCAM1^+^ cells in the neointima (arrows), NOTCH3^+^ cells in the neointima (arrowheads) and cells expressing both VCAM1 and NOTCH3 (asterisk) are marked. (*E*) The proportion of neointimal Confetti^+^ cells with specified marker expression at different time points after injury (*n* = 3 animals per time point). Bars show mean, error-bars SD. (*A*, *D*) Signals show VCAM1 (magenta), NOTCH3 (cyan), and Confetti (CFP: blue, RFP: red, YFP: yellow, and GFP: green) as indicated. Dashed lines outline the internal elastic lamina. L, lumen; P, plaque, M, media; NI, neointima.

We then spatially mapped the imVSMC-to-fcVSMC transition, using Myh11^Confetti^ mice where cell patches expressing the same fluorescent reporter protein represent clonally related VSMC-derived cells. *Notch3* expression was largely confined to fcVSMCs in both atherosclerosis and injury (*Figure 2C*; Supplementary material online, *Figure S2D*), whereas *Vcam1* was highly expressed by imVSMCs and down-regulated in fcVSMCs (*Figures 2C* and *3D*). Hence, VCAM1^+^NOTCH3^−^ cells highlighted the imVSMC state and VCAM1^+^NOTCH3^+^ represent cells at the cluster boundary (A-cluster 4-to-6 or I-cluster 3-to-6). VCAM1 and NOTCH3 expression was scored in Confetti^+^ cells in 26 individual clonal patches of VSMC-derived cells (16 plaques from three Myh11^Confetti^; *Apoe^−/−^* animals, *Figure 4A–C*; Supplementary material online, *Figure S5A*). VSMC clones containing cells with all marker combinations (VCAM1^+^NOTCH3^−^, VCAM1^+^NOTCH3^+^ and VCAM1^−^NOTCH3^+^ cells) were most frequently observed (42%; *Figure 4B*), showing that the progeny of a single VSMC can generate these three cell states. We did not observe VSMC clones containing only VCAM1^−^NOTCH3^+^ cells in any plaques, whilst VCAM1 was detected without NOTCH3 in 31% of clones (*Figure 4B*). This is consistent with the idea that VCAM1 induction is associated with VSMC entry into the plaque and that cells subsequently transition to the NOTCH3^+^ state.

To assess whether the local plaque environment impact the likelihood of generating the imVSMC and fcVSMC states, we quantified marker-expressing cells separately for the luminal and core regions of each VSMC clone. In keeping with previous observations,^20^ we found abundant luminal NOTCH3 expression by VSMCs, whereas VCAM1 expression was more prominent in the core region of VSMC clones (*Figure 4A* and *C*). Similar proportions of the three cell states were detected in the luminal region, suggesting this is the site of imVSMC-to-fcVSMC state transition. Together, these data show that the computationally inferred transition from imVSMC to fcVSMC state occurs *in vivo*, and that this represents formation of the fibrous cap in atherosclerotic plaques.

To more directly test whether lesional NOTCH3^+^ fcVSMC cells are generated from VCAM1^+^ imVSMCs, we leveraged the acute and reproducible kinetics of lesion formation in the carotid ligation injury model. All three states (VCAM1^+^NOTCH3^−^, VCAM1^+^NOTCH3^+^, and VCAM1^−^NOTCH3^+^) were also detected within single VSMC clones in injury-induced lesions (*Figure 4D*), however, the frequencies varied over time. Initially, almost all lesional VSMCs were VCAM1^+^NOTCH3^−^ (86%, 7 DPI, *Figure 4E*), but a substantial proportion of VCAM1^+^NOTCH3^+^ cells (19%) were observed in the growing intima at day 10–12. At 15 DPI, VCAM1^+^NOTCH3^−^, VCAM1^+^NOTCH3^+^, and VCAM1^−^NOTCH3^+^ states were detected at similar frequency (∼20% each; *Figure 4E*), whereas almost all lesional cells were VCAM1^−^NOTCH3^+^ at 28 DPI (87%; *Figure 4E*). For comparison, contractile gene re-expression was reported 2 weeks after injury.^31^ Together, these data demonstrate that VSMCs enter lesions in an imVSMC state and that clonal progeny of individual VSMCs transition to the fcVSMC state within injury-induced lesions. VSMCs in the medial layer of injured arteries expressed VCAM1 only at 7 DPI, NOTCH3 only at 15 and 28 DPI, and we did not detect VCAM1^+^NOTCH3^+^ cells at any time points (see Supplementary material online, *Figure S5B*), suggesting that the kinetics or underlying mechanisms may differ.

### 3.4 The thrombin receptor PAR1 is a candidate therapeutic target for promoting the fibrous cap-associated VSMC transition

**Figure 5:**
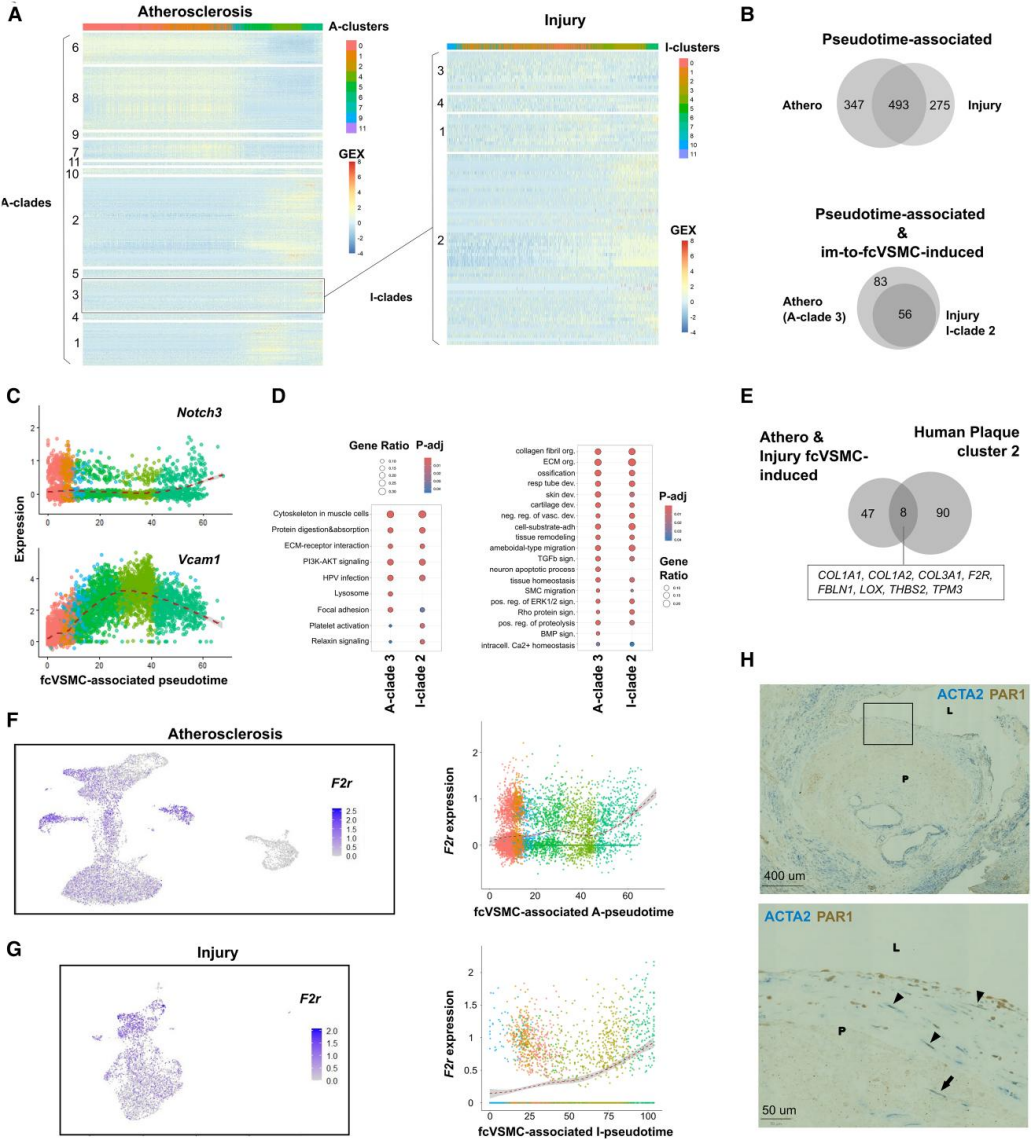
Identification of PAR1 as a candidate regulator of VSMC differentiation to a fibrous cap-associated VSMC state. (*A*) Heatmap of pseudotime-dependent genes [*P*-adj < 0.05, log(fold-change) > 0.25] for the fcVSMC-associated trajectory in atherosclerosis clustered into ‘A-gene clades’ (left), and A-clade 3 genes clustered into ‘I-gene clades’ by expression along fcVSMC-associated pseudotime in injury (right). Cluster affiliation (top) is shown using *Figures 2A* (A-clusters, left) and 3A (I-clusters, right) colour scales. Blue (low)-to-red (high) colour-scale shows scaled gene expression. (*B*) Overlap of all fcVSMC pseudotime-associated genes in atherosclerosis and injury (top) and intersection of A-clade 3 and I-clade 2 genes (lower panel). (*C*) *Notch3* and *Vcam1* expression along pseudotime for fcVSMC generation in injury. Dashed line shows a GAM. (*D*) Selected gene ontologies enriched in A-clade 3 and I-clade 2 genes (see Supplementary material online, *Tables S8*-*S11*). *P*-adj: cumulative hypergeometric probability testing with g:SCS correction. (*E*) Venn diagram for common fcVSMC-induced genes (I-clade 2) and genes induced in human fibrous cap-located spatial transcriptomics capture spots. The eight overlapping genes are listed. (*F, G*) *F2r* feature plots (left) and pseudotime-dependent expression in atherosclerosis (*F*) and injury (*G*). (*H*) Representative immunohistochemistry staining for ACTA2 and PAR1 in sections of human carotid plaques (*n* = 5). Panels show a large region of the plaque (top) and a magnified view of the boxed region (bottom). Signals for ACTA2 (blue) and PAR1 (brown) are shown as indicated. Examples of ACTA2 ^+^ PAR1^+^ cells in the cap (arrowheads) and ACTA2 ^+^ PAR1^+^ cells (arrow) in the core are shown. Scale bars = 400 µm (top), 50 µm (bottom). L, lumen; P, plaque, M, media.

To understand the regulatory mechanisms governing fcVSMC generation, we aimed to identify genes that are induced as cells undergo imVSMCs-to-fcVSMCs conversion. In total, 840 genes showed statistically significant pseudotime-dependent expression for the fcVSMC-generating trajectory in atherosclerosis [*P*-adj < 0.05, log(fold-change) > 0.25, *Figure 5A*]. These overlapped with pseudotime-associated genes for the equivalent trajectory in injury (59%; X^2^ test, *P* < 0.001; *Figure 5B*), further demonstrating the similarity of VSMC regulation in these models. Distinct pseudotime-dependent patterns were observed for *Vcam1* and *Notch3* (*Figure 5C*).

Hierarchical clustering of pseudotime-associated genes by expression pattern highlighted a gene subset (A-clade 3, boxed in *Figure 5A*) that is specifically up-regulated at the imVSMC-to-fcVSMC transition. A similar pseudotime-dependent induction was observed for 56 of the 84 A-clade 3 genes in the injury model (I-clade 2; *Figure 5A* and *B*). Pathway analysis indicated consistent enrichment of ECM organization, migration and adhesion for these ‘fcVMSC induced genes’ and highlighted signalling pathways, including TGF-beta, as potential regulators (*Figure 5D*). We focused on I-clade 2 genes, hypothesizing that the acute, accelerated kinetics of lesion formation in this model would enrich for genes that drive fcVSMC differentiation, rather than being expressed as a consequence of the transition.

The relevance to fibrous cap formation in human atherosclerosis was assessed using spatial transcriptomics. To capture the regulatory mechanisms driving ongoing fibrous cap development, we analysed an aortic sample with an early stage, fibrous cap-containing plaque (see Supplementary material online, *Figure S6*). A cluster of capture spots (human plaque [HP]-cluster 2, localized to the luminal edge of the lesion) had abundant levels of contractile markers and low expression of immune cell markers (*CD68*, *CD163*, and *CD86)* suggesting that these represent the fibrous cap cluster that contained mainly VSMCs. Eight genes of the 56 fcVSMC-induced genes were among the 90 marker genes for HP-cluster 2 (X^2^ test, *P* < 0.001), including fibrillar collagens^33^ (*COL1A1, COL1A2, COL3A1), FBLN1, LOX, TPM3, THBS2*, and *F2R*; *Figure 5E*; Supplementary material online, *Figure S6C*–*E*).

**Figure 6:**
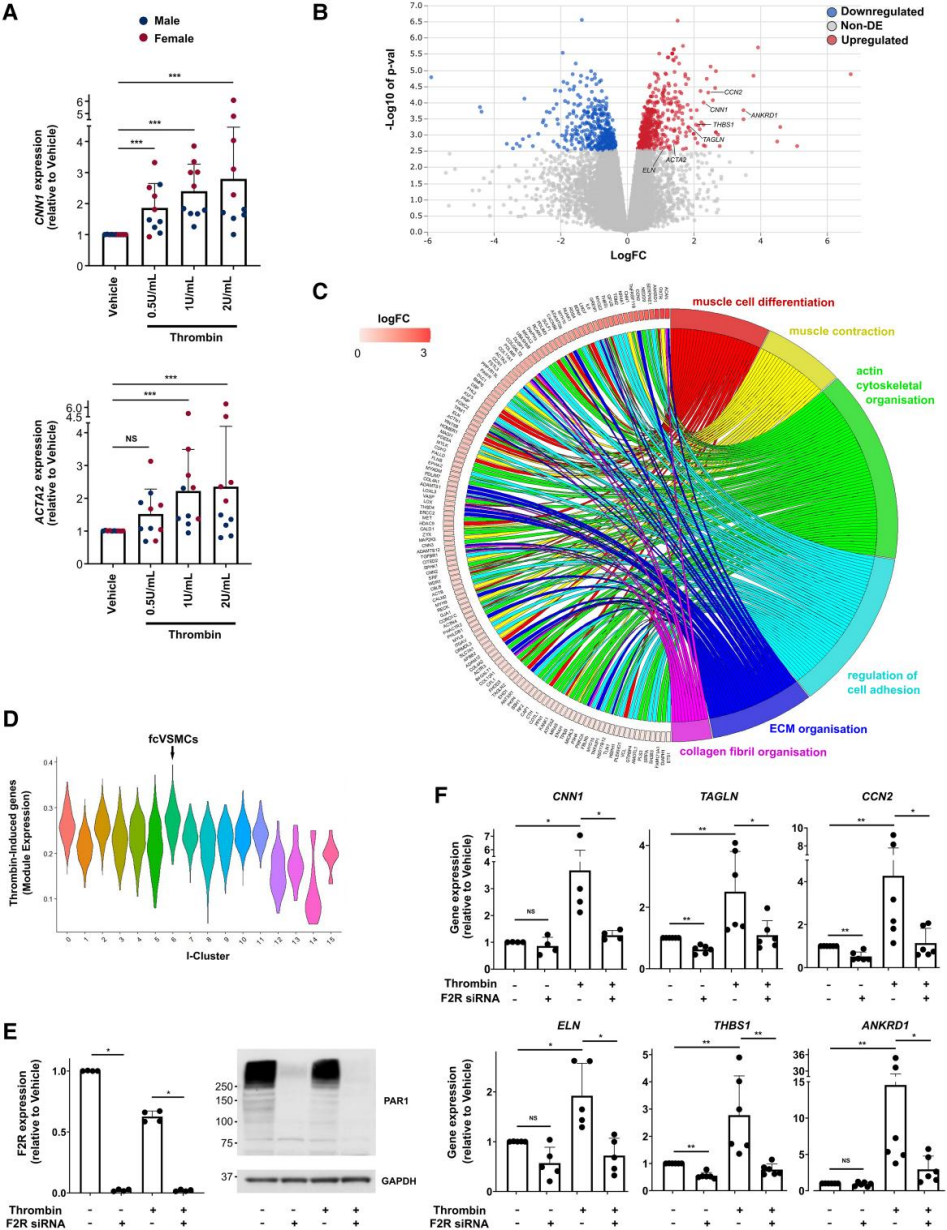
PAR1 stimulation induces a transcriptional pattern associated with fibrous cap cells. (*A*) Transcript levels (qRT-PCR) of contractile genes *CNN1* and *ACTA2* in primary human VSMC (hVSMC) isolates from different donors (*n* = 5 male, blue, and 5 female donors, red) after 24 h vehicle or thrombin treatment (0.5, 1, or 2 U/mL). (*B*) Volcano plot showing differential gene expression between thrombin-treated (2 U/mL) vs. vehicle control samples (*n* = 3 female donors). Differentially expressed genes are coloured (*P*-adj < 0.05, red: induced by thrombin, blue: reduced by thrombin) and genes of interest indicated. See Supplementary material online, *Table S12*. (*C*) VSMC- and ECM-associated GO terms enriched in thrombin-induced genes and associated differentially expressed genes [white-red scale, log(fold-change)]. (*D*) Violin plot showing expression of thrombin-induced gene orthologues across the injury cell clusters. The arrow marks fcVSMC I-cluster 6. (*E*) *F2R* transcript levels (left, normalized to vehicle treated, *n* = 4) and western blot (right; molecular weight in kDa, *n* = 3) in primary hVSMCs ± thrombin with *F2R*-targeting (+) or control siRNA (−). (*F*) Transcript levels of candidate target genes in thrombin-treated hVSMCs relative to vehicle-treated samples with *F2R*-targeting (+) or control siRNA (−). *n* = 4–6. (*A*, *E*, *F*) Bars show mean ± SD relative to vehicle. *P-adj < 0.05, **P-adj < 0.01, ***P-adj < 0.001, NS: not significant (Mann–Whitney with Bonferroni-correction, following Kruskal–Wallis with *P* < 0.05). Replicates are hVSMC lines from different donors.

We selected *F2R*, which encodes the thrombin receptor or protease-activated receptor 1 (PAR1), for further analysis. *F2r* transcripts were detected at high levels in fcVSMC clusters in both atherosclerosis and injury models, with low expression in A-cluster 7 (termed minor VSMC^12^) FB-like A-cluster 8 cells, and imVSMCs (I-cluster 3) (*Figure 5F* and *G*). Immunostaining in human atherosclerosis revealed abundant PAR1-expressing ACTA2^+^ VSMCs in human plaque fibrous caps, and PAR1 protein was also detected at lower levels in the core (*Figure 5H*). Previous investigations have shown that thrombin promotes VSMC proliferation,^34,35^ but a role in VSMC re-differentiation and fibrous cap generation has not been investigated. We therefore assessed whether PAR1 functionally affects the imVSMC-to-fcVSMC transition, and thus potentially fibrous cap formation in primary human VSMC isolates. We observed a dose-dependent, statistically significant increase in *CNN1* and *ACTA2* expression in response to physiologically relevant thrombin concentrations (*Figure 6A*). Interestingly, the effect of thrombin was more profound in VSMCs isolated from female compared to male donors (*Figure 6A*; *P* = 0.008, Mann–Whitney *U* test).

To comprehensively evaluate the transcriptional state induced by PAR1 stimulation, we profiled gene expression in thrombin-treated and untreated cells using RNA-seq. We confirmed up-regulation of contractile VSMC genes (including *CNN1, ACTA2*, and *TAGLN*) in thrombin-treated vs. control cells, and also detected increased expression of factors that have been associated with VSMC differentiation and/or fibrous cap development (*Figure 6B*). For example, connective tissue growth factor (CCN2) is involved in maintenance of the contractile VSMC state and is expressed in the fibrous cap^36^; elastin (ELN) is a key ECM component of the fibrous cap and also functions to maintain VSMC contractility^37^; Thrombospondin 1 (THBS1) has been implicated as a key factor involved in fibrous cap formation^38^; and Cardiac ankyrin repeat protein (ANKRD1) is expressed by VSMCs of the fibrous cap.^39^ Systematic analysis showed that the 492 thrombin up-regulated genes (*P*-adj < 0.05) were enriched for GO-terms associated with muscle cell differentiation, contraction, and focal adhesion as well as ECM organization, including collagen fibril organization, which is a key component of fibrous cap formation^33^ (*Figure 6C*; Supplementary material online, *Table S12* and *S13*). A gene module comprising murine orthologues of thrombin-induced genes exhibited peak expression in the fcVSMC population identified in injured mouse arteries (I-cluster 6; *Figure 6D*). This suggests an overall induction of a fibrous cap-associated VSMC state by thrombin.

Thrombin-induced genes were also stimulated in response to another PAR1 protease, Factor Xa, although this did not reach statistical significance for all genes (see Supplementary material online, *Figure S7A*). To directly test whether protease-induced expression of contractile and fibrous cap-relevant genes was mediated by thrombin receptor stimulation, we targeted PAR1 activity using siRNA (*Figure 6E*; Supplementary material online, *Figure S7B*), which blunted or abolished thrombin-induced expression of *CNN1, TAGLN, CCN2, ELN, THBS1*, and *ANKRD1* (*Figure 6F*). A PAR1-specific small molecular inhibitor, vorapaxar, also blocked thrombin-mediated gene induction (see Supplementary material online, *Figure S7C*). Interestingly, baseline expression of *TAGLN*, *CCN2* and *THBS1* was reduced by PAR1 knockdown, suggesting thrombin-independent regulation of these genes by PAR1 (*Figure 6F*). Together, these data indicate that thrombin receptor activation in VSMCs leads to induction of fcVSMC-associated genes.

## 4. Discussion

Here, we provide evidence that a novel VSMC sub-population (that we term fcVSMCs) is derived from VSMCs expressing a previously described intermediate modulated, or imVSMC, state, is present in the fibrous cap of plaques, and can be modelled in injury-induced lesions: (i) the expression of key fibrous cap-associated genes by fcVSMCs in both atherosclerosis and injury, (ii) trajectory analysis consistently predicts that fcVSMCs are derived from imVSMCs, (iii) co-detection of imVSMCs and fcVSMCs markers in clonally related cells in atherosclerotic lesions, (iv) initial absence of intimal NOTCH3-expression after injury, and (v) the fcVSMC marker, NOTCH3, shows abundant expression in the fibrous cap. We also identify candidate regulators of the proposed intermediate-to-fcVSMC transition, including *F2R*-encoded PAR1 and provide evidence suggesting that PAR1 stimulates the fibrous cap state. Finally, we show that PAR1 stimulation by thrombin in primary human VSMCs induces fibrous cap VSMC markers and a transcriptional profile matching that of fcVSMCs. We therefore suggest that PAR1 stimulation in VSMCs could promote fibrous cap formation.

The notion that an imVSMC state gives rise to fibrous cap VSMCs aligns with previous models. In particular, *Ly6a*/SCA1-expressing VSMCs are present prior to disease and have increased proliferative capacity,^13^ similar to the SCA1^+^ cells isolated from lesions.^12^ It is therefore conceivable that cells in an imVSMC state invade lesions and expand clonally to generate all VSMC-derived plaque cells, including those comprising the fibrous cap. Other groups have shown that manipulation of factors that promote the imVSMC state disrupt fibrous cap formation.^18,19^ NOTCH loss-of-function was required for VSMC plaque entry, whereas NOTCH3 reactivation is required for fibrous cap formation^20^ and NOTCH signalling is known to promote the contractile VSMC phenotype.^40^ This is consistent with our findings (i) that fcVSMCs up-regulate *Notch3*, (ii) our confirmation of NOTCH3 expression in fibrous cap VSMCs and delayed NOTCH3 expression when compared to VCAM1. Our findings are also consistent with dual lineage tracing experiments showing that *Lgals3*-expressing modulated cells generate all lesional VSMCs, including those in the fibrous cap at early atherogenesis.^15^ Alternative pathways may also contribute to fibrous cap formation, and could explain the presence of non-Lgals3-lineage cells at later plaque stages.^15^

PAR1 is expressed by VSMCs and thrombin is a well-known mitogen of these cells *in vitro*, putatively via PAR1.^34^^,35^ This proliferation-stimulating effect may exacerbate the response to vascular injury.^41^ VSMC proliferation has been detected in the fibrous cap^10^ and it is generally considered to indicate plaque stability, however, the role of PAR1 in fibrous cap formation has not been studied. Our finding that PAR1 stimulation induces expression of contractile genes in cultured VSMCs is consistent with studies in rat VSMCs^42^ and analysis of published microarray data from human aortic VSMCs after shorter thrombin treatment.^43^ Furthermore, previous work has shown that the PAR1 target CCN2, was also induced by thrombin in rat VSMCs.^44^

PAR1 is also expressed on platelets and by endothelial cells. Systemic inhibition of either thrombin or PAR1 attenuated atherosclerosis progression and even promoted features of plaque stability, due to effects on endothelial function, proinflammatory signalling and immune cell infiltration.^41,45–48^ Thus, cell type specific deletion of PAR-1 is needed to directly assess VSMC-specific effects in disease and, specifically, fibrous cap formation in atherosclerosis lesions. This could also hamper therapeutic translation of these findings. However, the identification of PAR1 as a candidate regulator of fibrous cap formation could therefore have several implications for the management of atherosclerosis. First, anticoagulants used to treat patients with atherosclerotic disease inhibits PAR1 (vorapaxar) or thrombin directly.^49,50^ Given the fibrous cap-inducing effect of VSMC-expressed PAR1 suggested here, vorapaxar treatment could have an unintended negative effect on plaque stability. Secondly, the induction of fcVSMC-associated genes by thrombin was more prominent in female than male VSMCs, and could contribute to the sex differences in plaque biology, particularly, the greater frequency of thin or ruptured fibrous caps in male patients.

Our observation that VSMC states induced by injury overlap those observed in atherosclerosis suggests that transitions to plaque VSMC states is an inherent characteristic of VSMCs in different physiological settings. Indeed, there are many commonalities between the two disease models. Both atherosclerotic and injured arteries show endothelial activation and a key role of inflammation, suggesting overlap in triggering mechanisms. The absence of a fibrous cap and high cholesterol in the injury model are notable differences. Recent evidence has suggested that plaque cholesterol induces VSMC responses similar to those of inflammation,^51^ which may explain the remarkable similarities of these vascular disease models. VSMC proliferation, migration and oligoclonal contribution to lesions is observed in atherosclerosis, aneurysm, and injury, and the substantial overlap in gene regulation highlighted here has also been found in other studies. For example, recent scRNA-seq analyses indicated that imVSMCs are present in both atherosclerotic and injured arteries.^13,32,52^ Our observation that an fcVSMC population is present in both models indicates that aspects of fibrous cap formation are analogous to VSMC re-differentiation in injury-induced lesions, although a fibrous cap structure is not formed. Importantly, this implies that the carotid ligation injury model, which is acute and has reproducible kinetics compared to experimental atherosclerosis, could be used to model the generation of fibrous cap cells. For instance, manipulation of candidate regulators, such as PAR1, could be used to screen factors for effects on the proportion of fcVSMCs in neointimal lesions at well-characterized time points.

The identification of a VSMC transition associated with fibrous cap formation provides exciting new insight into the mechanisms underpinning plaque stability. We propose that deeper understanding of this transition and its regulation could provide new drug targets that could be used for promoting fibrous cap formation and thus mitigation of plaque rupture.

Translational perspective Identifying regulators of the VSMC transition that generates fibrous cap cells could enable development of new interventions to stabilize atherosclerotic plaques and mitigate acute cardiovascular events resulting from plaque rupture.

## Supplementary Material

cvaf112_Supplementary_Data

## Data Availability

Bulk (GSE274393), spatial (GSE274572), and single-cell RNA-seq datasets generated in this study (GSE274572) have been deposited, and VSMC-lineage-labelled plaque cell profiles from HFD-fed animals (GSE155513) are available from the gene expression omnibus (GEO). Source data are available upon request.
