# Supplementary material for: Delineation of a thrombin receptor-stimulated vascular smooth muscle cell transition generating cells in the plaque-stabilizing fibrous cap
Source: Cardiovasc Res. 2025 Jun 27;121(9):1359–72. doi: 10.1093/cvr/cvaf112 (PMC12352313; doi:10.1093/cvr/cvaf112)
Supplement: cvaf112_Supplementary_Data [file cvaf112_supplementary_data.zip › Taylor_etal_Supplemental_Correction180625.docx]

**Supplementary Information**

**Delineation of a thrombin receptor-stimulated** **vascular smooth muscle cell transition generating cells in the plaque-stabilising fibrous cap**

James CK Taylor^1,^ *, Matthew D Worssam^1^, Sebnem Oc^1, 2^, Jordi Lambert^1^, Krishnaa T Mahbubani^3^, Kirsty Foote^1^, Allie Finigan^1^, Yee-Hung Chan^1^, Nichola Figg^1^, Murray C H Clarke^1^, Martin R Bennett^1^, Helle F Jørgensen^1,^ *.

Supplementary Figures S1-S7:

Figure S1: Atherosclerosis scRNA-seq analysis

Figure S2: Characterisation of injury (11 DPI) scRNA-seq dataset

Figure S3: Characterisation of integrated dataset shown in Figure 3B

Figure S4: Integration of injury scRNA-seq experiments

Figure S5: Quantification of medial VCAM1 and NOTCH3 staining following injury

Figure S6: Spatial transcriptomics of human plaque

Figure S7: Analysis of PAR1-regulated gene expression

Supplementary Methods

Supplementary References

Supplementary Tables S1-S12 (provided as separate spreadsheets):

Table S1 Summary of plaque features

Table S2 RT-qPCR primer sequences

Table S3 scRNA-seq data analysis parameters

Table S4 Atherosclerosis (A)-cluster markers

Table S5 A-cluster 4 *vs*. A-cluster 6 differential expression

Table S6 GO-terms enriched in fcVSMC *vs.* imVSMC genes in atherosclerosis

Table S7 Injury (I)-cluster markers

Table S8 Atherosclerosis (A)-pseudotime-associated genes

Table S9 GO-terms enriched in A-clade 3 (84 genes)

Table S10 Pseudotime-associated gene expression clades in injury for A-clade 3

Table S11 GO-terms enriched in I-clade 2 genes (56 genes)

Table S12 Genes with differential expression in thrombin-treated cells *vs.* controls

Table S13 GO-terms enriched in genes induced by thrombin *vs.* controls

**Supplementary Figures**

**
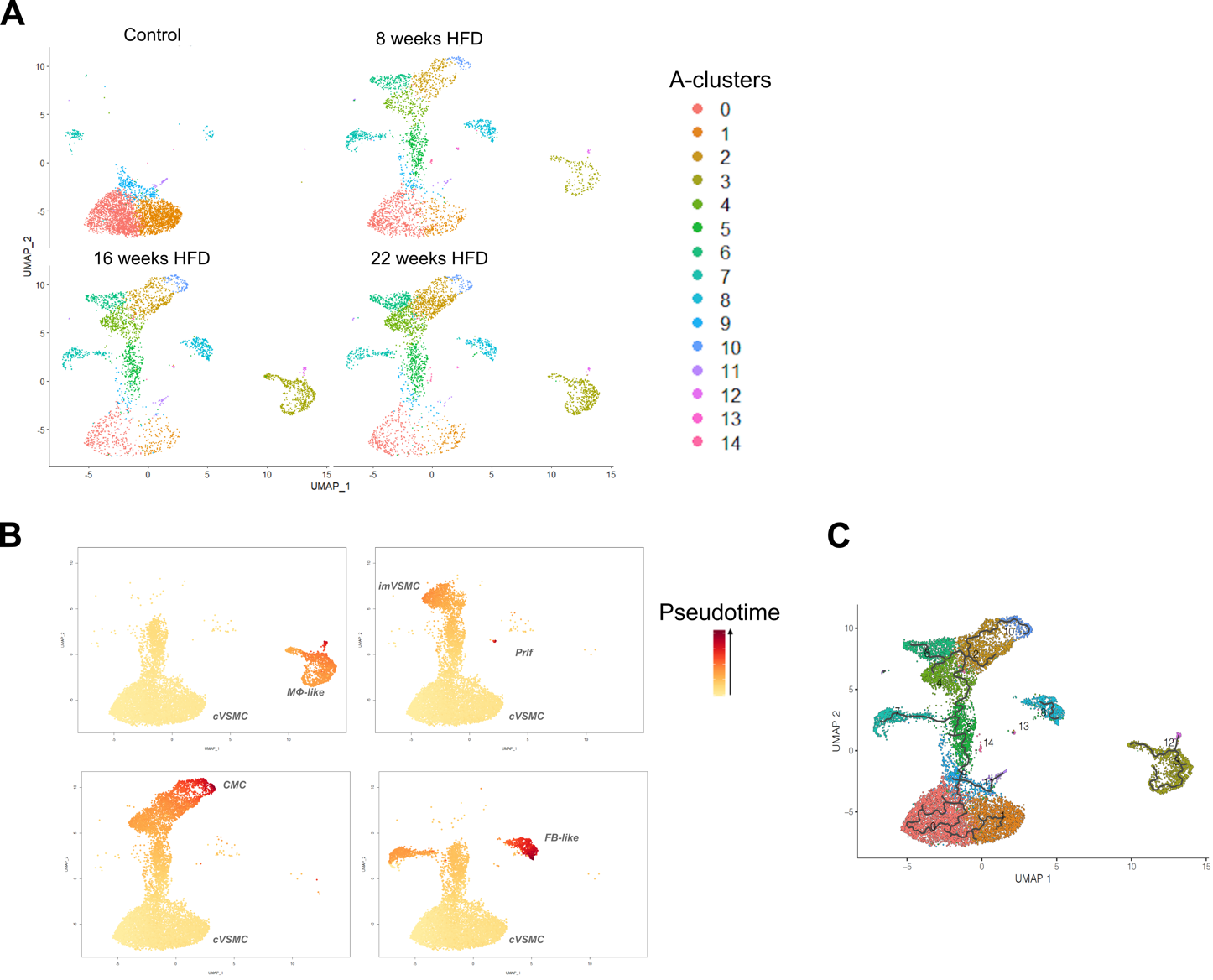
**

**Supplementary Figure S1. Atherosclerosis scRNA-seq analysis.** (***A***) Uniform Manifold Approximation and Projection (UMAP) with cell cluster map split by vessel health status/high fat diet (HFD) timepoint for scRNA-seq analysis of VSMC-lineage label positive cells from atherosclerotic arteries (GSE155513) of Myh11-ZsGreen/Apoe-/- animals after 8, 16 and 22 weeks of HFD feeding and healthy control Apoe^+/+^ carotid arteries. (***B***) Trajectories inferred in atherosclerosis dataset that generate macrophage-like (top left), proliferating (top right), chondro, CMC (lower left) and fibroblast-like, FB (lower right) VSMC states. The trajectory generating the fcVSMC state is shown in main figure 2E. Only cells that are part of each trajectory is shown, and pseudotime progression is indicated using a yellow-to-red colour code. (***C***) Trajectories predicted using Monocle3.

**Supplementary Figure S2. Characterisation of injury (11 DPI) scRNA-seq dataset.** (***A***) Dot plot showing expression of cell state markers and the eYFP lineage reporter in I-clusters using a scale from grey (low) to blue (high) and the percentage of cluster cells with detected marker expression indicated by dot size. Clusters comprising the main, contiguous cell population are marked. cVSMC: contractile VSMC; imVSMC: intermediate modulated VSMC; ECM: extracellular matrix; CMC: chondromyocyte; FB: fibroblast; MΦ: macrophage; Prlf: proliferation. **Lgals3* is also expressed by imVSMCs. (***B***, ***C***) Trajectory inference analysis using Monocle3 (***B***) or Partition-based graph abstraction (PAGA) analysis, where line thickness indicates degree of transcriptional similarity between clusters (***C***).

**Supplementary Figure S3. Characterisation of integrated dataset shown in Figure 3B.** (***A***) Uniform Manifold Approximation and Projection (UMAP) showing integrated dataset clustering for injury (panel ***A***), atherosclerosis (Figure 2A), and healthy control carotid arteries scRNA-seq profiles. (***B***) Proportion of cells from individual datasets that contribute to integrated dataset cell clusters. (***C***) Dot plot showing expression of selected genes in integrated dataset cell clusters using a grey (low) to blue (high) scale and the percentage of cells with detected marker expression for each cluster indicated by dot size. (***D***) UMAP feature plots showing expression levels for cVSMC (*Myh11),* imVSMC (*Vcam1, Lgals3)* and fcVSMC genes (*Notch3*), split by dataset, using grey (low) to blue (high) scales.

**Supplementary Figure S4. Integration of injury scRNA-seq experiments.** Uniform Manifold Approximation and Projection (UMAP) showing integration of 11 DPI experiment 1 (Exp1) and Exp2 together (***A***) or split by experiment (***B***). In panel ***B***, I-cluster numbers from experiment 1 are shown.

**Supplementary Figure S5. Analysis of VCAM1 and NOTCH3 expression in mouse models with clonal VSMC lineage tracing.** (***A***) Immunostaining of plaque-containing artery of Myh11-CreERt2/Confetti/Apoe-/- animal after 11 weeks high fat diet. Signals for GFP (green, to detect lamina autofluorescence), Confetti, VCAM1 (magenta), NOTCH3 (cyan), and DAPI (white) are shown as indicated. Plaque VSMCs (red lineage label) expressing VCAM1 only, NOTCH3 only or co-expressing VCAM1 and NOTCH3 are detected. In non-remodelled parts of the artery, medial VSMCs display widespread NOTCH3 expression and low levels of VCAM1, similar to what was reported in healthy vessels previously (doi: 10.1093/cvr/cvac138, doi: 10.1161/ATVBAHA.120.315627). This contrasted with substantially reduced NOTCH3 levels in the media under lesions, which also displayed higher VCAM1 expression. (***B***) The proportion of medial Confetti+ cells in diseased artery regions with specified marker expression at indicated timepoints after injury of Myh11-CreERt2/Confetti mice (n=3 animals per timepoint).


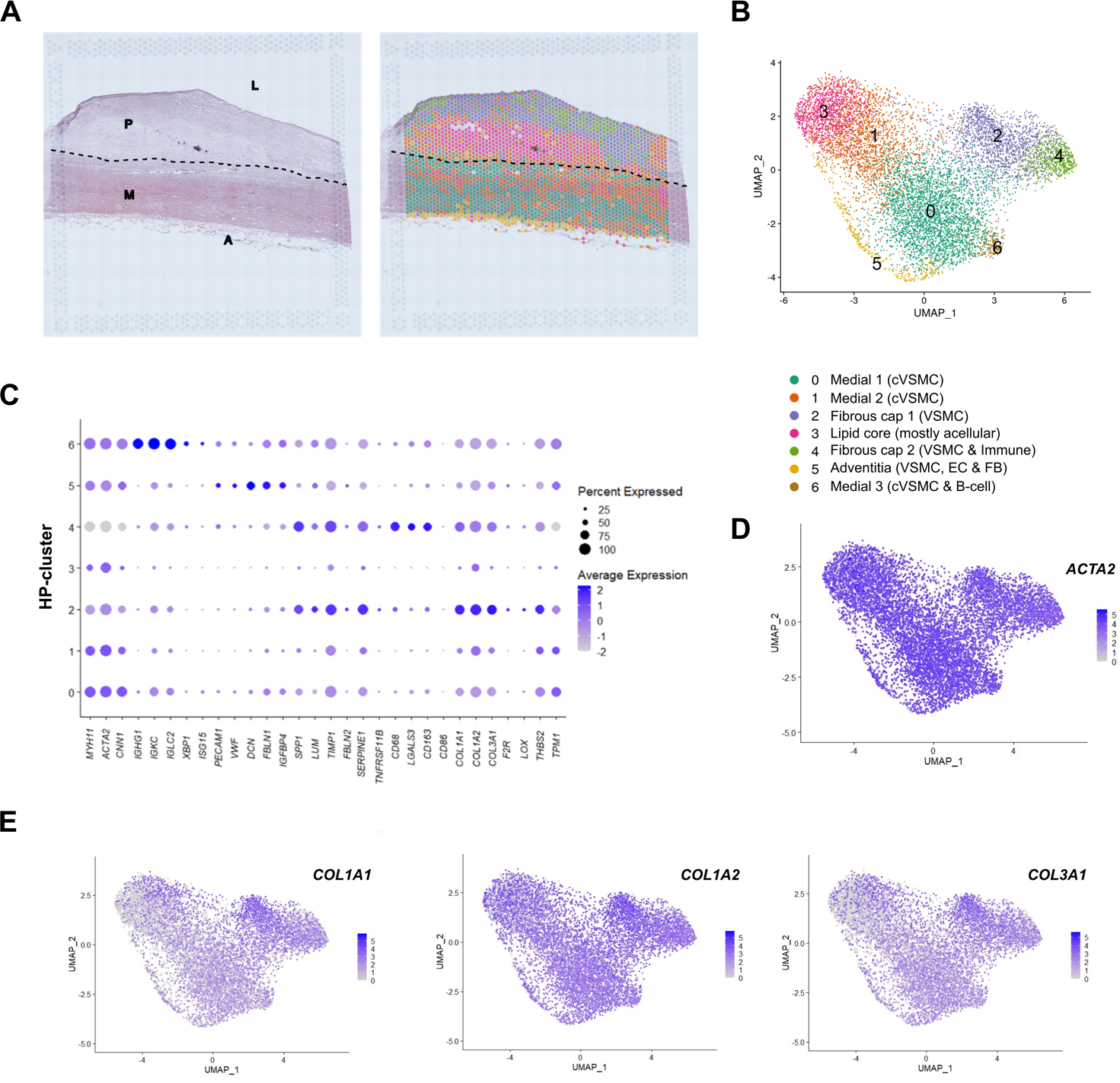


**Supplementary Figure S6. Spatial transcriptomics of human plaque.** (***A***) Haematoxylin and eosin-stained section labelled by tissue regions (left) or overlaid with capture spot cluster annotations [human plaque (HP)-cluster 0-6, right] or. L, lumen; P, plaque, M, media; A, adventitia. (***B***) Uniform Manifold Approximation and Projection (UMAP) of capture spots coloured by HP-cluster affiliation. (***C***) Dot plot showing expression of cell type markers in clusters using a scale from grey (low) to purple (high). HP-cluster 0 and 1 are located in the media and expressed contractile VSMC markers at high levels (*MYH11, ACTA2, CNN1;* Supplementary Figure 5). Low-abundance HP-cluster 6 spots in the media expressed contractile VSMC markers and, unexpectedly, immunoglobulin genes (*IGHG1, IGKC, IGLC2*), indicating VSMC and, unexpectedly, B cell presence. B cells are generally not located in the medial layer, but immunoglobulin deposits have been detected in the media of atherosclerotic arteries, suggesting B cell presence here is possible (doi:10.4049/jimmunol.1202870). HP-cluster 5 spots localised to the adventitia and expressed a mix of VSMC, endothelial (*PECAM1, VWF*) and fibroblast (*DCN, FBLN1, IGFBP4*) markers. HP-cluster 3 spots were characterised by poor QC parameters and predominantly resided within the lipid core, a largely acellular region. HP-cluster 2 and 4 were located in the fibrous cap and expressed markers of modulated VSMCs (*SPP1, LUM, TIMP1, FBLN2, SERPINE1, TNFRSF11B)*. Immune cell markers were also abundantly expressed in HP-cluster 4 (*CD68, CD163, CD86*), but these were detected at lower levels in HP-cluster 2. HP-cluster 2 is the fibrous cap cluster that contained mainly VSMCs. The percentage of capture regions within clusters with detected marker expression is indicated by dot size. (***D****,* ***E***) UMAP feature plots showing expression of *ACTA2* (***D***) and fibrillar collagen (***E***).


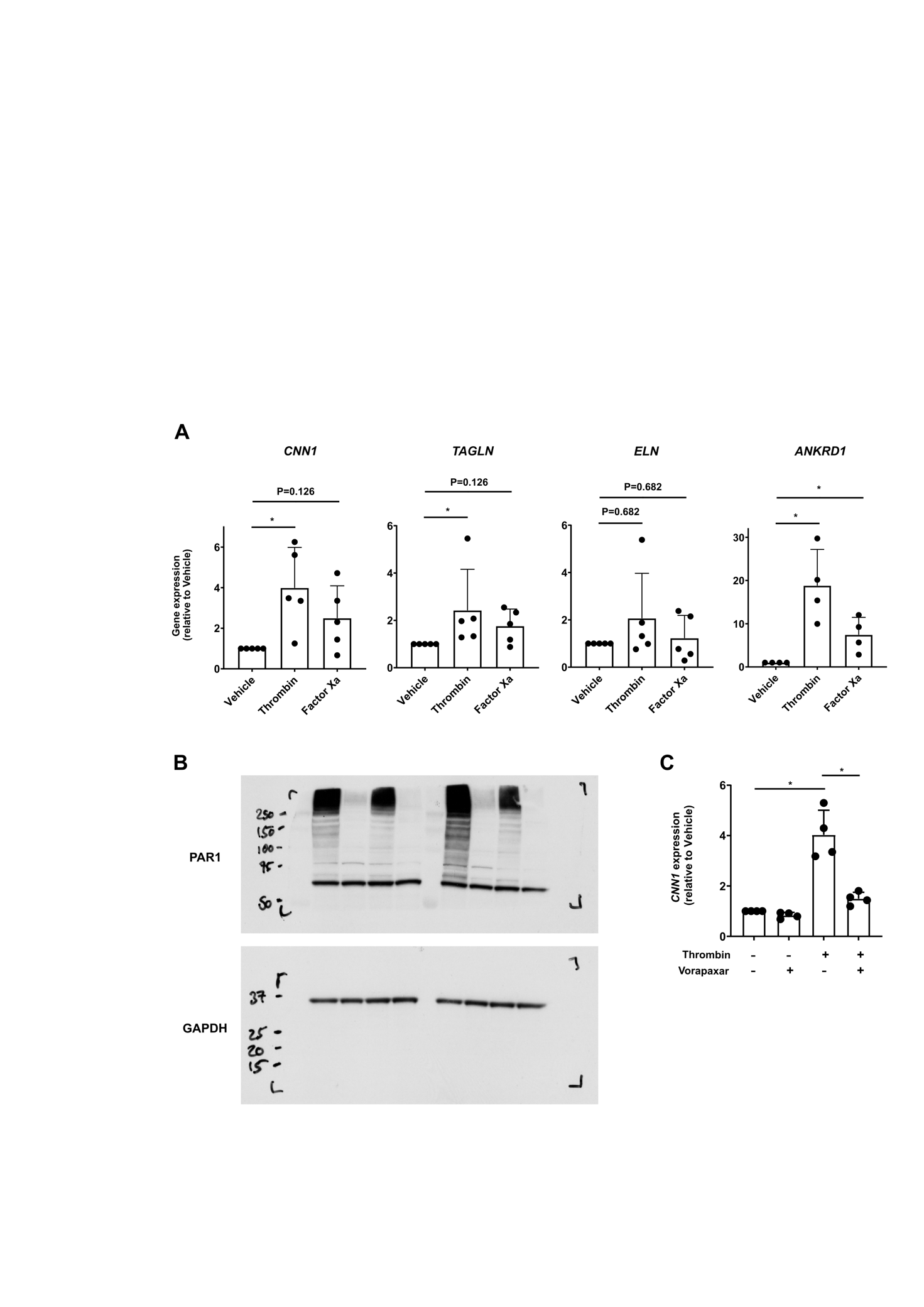


**Supplementary Figure S7. Analysis of PAR1-regulated gene expression.** (***A***) Expression levels of *CNN1*, *TAGLN*, *ELN* and *ANKRD1* in human VSMCs treated with Factor Xa or Thrombin, relative to vehicle-treated samples. n=5 independent donors (female). (***B***) Uncropped version of western blot for PAR1 (top) and GAPDH (right). MW mark lanes (1+6) with molecular weight markers, the sizes are shown at the left (kDa). Lanes 2-5 are presented in panel 6E (2: -Thrombin, siNTC; 3: -Thrombin, siF2R; 4: +Thrombin, siNTC; 5: +Thrombin, siF2R), lanes 7-10 are equivalent samples from a replicate experiment (n=3). The membrane was cut prior to antibody incubation to allow parallel antibody probing; the outline of membranes are marked. (***C***) Expression levels of *CNN1* in thrombin-treated human VSMCs relative to vehicle-treated samples with and without co-treatment with vorapaxar. Bars show mean±SD. n=4 independent donors lines (female). *P-adj<0.05 (Mann-Whitney with Bonferroni multiple testing correction, Statistical testing was done between groups).

## Supplementary Methods

## Animals and procedures

All experiments were carried out according to the UK Home Office regulations under PPLs P452C9546 and PP7513347 and were approved by the University of Cambridge Ethical Reviews Committee. VSMC labelling was achieved using Myh11-CreER^T2^; Rosa26-Confetti (Myh11^Confetti^) and Myh11-CreER^T2^; Rosa26-eYFP (Myh11^eYFP^) mice aged 6-8 weeks by 10 intraperitoneal injections of 1 mg tamoxifen (Sigma) dissolved in corn oil over 2 weeks. Animals were rested for at least 7 days before inducing disease. Surgery was performed as described previously^1^. Briefly, Myh11^Confetti^ and Myh11^eYFP^ animals were subcutaneously administered pre-operative analgesic (~0.1 mg/kg body weight, buprenorphine), anaesthetised with 2.5-3% isoflurane by inhalation (1.5 L/min) and the left carotid artery tied off with 6-0 silk suture. Lineage-labelled Myh11^Confetti^; *Apoe^-/-^* mice were fed a high fat diet (HFD, Special Diets Services, containing 21% fat and 0.2% cholesterol) for the length of time indicated. Animals were culled at the indicated timepoint after surgery by CO_2_ asphyxiation and perfused with phosphate-buffered saline (PBS) prior to tissue removal. Mouse arteries were dissected free from adipose and connective tissue before experiment-specific processing (as described below).

## Single-cell RNA-sequencing (scRNA-seq) data generation

Datasets of lineage-labelled VSMCs from healthy and injured mouse carotid arteries were generated as described^2^ after pooling cells from 5 Myh11^eYFP^ animals 11 days post-ligation or 2 control Myh11^eYFP^ animals that did not undergo surgery. After carotid artery harvest and dissection, arteries were incubated for 6 minutes in collagenase type IV (1 mg/mL, Life Technologies) and porcine pancreatic elastase (1 U/mL, Worthington) in DMEM. After removing the adventitia, the medial samples from each animal cohort were pooled and digested in collagenase type IV (2.5 mg/mL, Life Technologies) and porcine pancreatic elastase (2.5 U/mL, Worthington) in DMEM for 90 minutes. Medial cell suspensions were filtered through a 40 µm filter and singlet cells expressing eYFP isolated by fluorescence-activated cell sorting (FACS, Aria-Fusion flow cytometer, BD Bioscience) into PBS with 0.05% BSA and 5 µL/mL recombinant RNAse inhibitor (Sigma). Isolation of single cells was verified with a haemocytometer. Sorted cells (10,000) were pelleted, resuspended in 47 µL of PBS with 0.05% BSA and loaded for Drop-Seq using the 10X Chromium system. Amplified cDNA libraries were sequenced on a HiSeq 4000 system.

## scRNA-seq data analysis

Raw sequencing reads were processed and aligned to the GRCm38 mouse genome (+eYFP cDNA/ORF sequence) through the 10X Genomics Cell Ranger pipeline (v3.1.0). Datasets for Myh11 promoter-driven, lineage-labelled VSMCs in a mouse atherosclerosis model have been published previously^3^ and gene-count matrices were downloaded from the gene expression omnibus (GEO, accession number GSE155513). All datasets were analysed using the R or Python programming language. Key package version numbers, dataset-specific quality control (QC) parameters, normalisation method and numbers of principal components (PCs) used for the analysis are indicated in Supplementary Table 1. For the mouse atherosclerosis dataset, QC parameters defined in the original publication^3^ were used. For cells that passed the QC criteria, expression data was normalised using either the *NormalizeData* or *SCTransform* (described previously^4^) function in Seurat^5^. The major principal components (PC’s), identified using a combination of the Elbow method and JackStraw function, were used for Louvain clustering and Uniform Manifold Approximation and Projection (UMAP). For comparison of cell states between conditions, datasets were integrated using the FindIntegrationAnchors and IntegrateData functions in Seurat^5^. The resolution was validated by assessing cluster boundaries using the sleepwalk package^6^ (CRAN). Differential gene expression between clusters was determined using the FindAllMarkers function in Seurat. Significance was determined by Wilcoxon rank sum test [FDR adjusted P-value (P-adj)<0.05] and differentially expressed gene thresholds of log_2_ fold-change (log_2_FC)>0.5 and minimum 25% of expressing cells within a cluster. Integration of healthy mouse carotid and atherosclerosis datasets was performed with the standard Seurat workflow using the FindIntegrationAnchors and IntegrateData functions (first 30 PCs used).

Trajectory analysis using the Slingshot package^7^ (Bioconductor) was was done after converting the Seurat object to a SingleCellExperiment (SCE) object using the SCE package^8^ (Bioconductor) using the same number of PCs used for dimensionality reduction and clustering. The cell cluster showing the highest contractile VSMC gene expression was selected as the origin for the analysis. Differential expression across the trajectory path of interest was performed with Generalised Additive Modelling (GAM) using the gam package^9^ (CRAN). The 3000 highly variable genes (HVGs) were used as input for this analysis. A GAM (with a Loess term for pseudotime) was fitted for each gene (using the gam function) and significance for pseudotemporal-dependent expression assessed (FDR-corrected P-adj<0.05). A magnitude threshold for the difference between the modelled maximum and minimum values in a log scale over trajectory pseudotime of logFC>0.25 was then applied for genes with significant pseudotime-dependent expression. The genes that passed these criteria were grouped into gene “clades” with similar expression patterns based on Pearson correlation distance and the complete linkage method, which were plotted in a heatmap over trajectory path pseudotime (all performed using the pheatmap package^10^ (CRAN). The optimal number of clades was determined using the Elbow method.

Partition-based graph abstraction (PAGA^11^) was performed on the injury dataset using functions available within Scanpy^12^ for Python. The package anndata2ri^13^ and associated extensions were used to interface R and Python languages. This was used to convert the injury dataset SCE (R) object into an AnnData (Python) object. From here, dimensionality reduction was performed as before using equivalent functions in the Scanpy package. The cluster labels obtained in the original Seurat analysis were retained. PAGA was then run using the sc.pl.paga function and an edge connectivity threshold of 0.35 was the highest value that permitted a connected structure of the data. The sc.pl.paga_compare function was used to visualise cell and cluster connectivity projections over UMAP plots generated with initial PAGA positions taken as additional input.

Monocle trajectory analysis was performed by using *monocle3*. Following the conversion of Seurat objects to Monocle3 objects (*cell_data_set* function), cells were grouped (*cluster_cells* function) into 16 cell clusters (resolution=6e-4) for atherosclerosis and 25 clusters (resolution=9e-3) for injury datasets. Then, lineages were inferred (*learn_graph* function) and cells were ordered (*order_cells* function) selecting root cell regions were based on contractile VSMC gene expression.

Ligand prediction was done using NicheNet (v2.2.0) using a sender-agnostic approach to identify potential ligands inducing the I-cluster 6 VSMC state. The default ligand-receptor network was used and ligands with detection of associated receptor transcripts in at least 5% of I-cluster 6 cells were considered. I-cluster 6 marker genes (p<0.05, |log_2_FC|>0.25) were included as target genes, with all I-cluster 6 expressed genes as background. The top five ligands, ranked by predicted ligand strength (area under the precision-recall curve), were selected and the regulatory potential of their top 100 gene targets assessed.

## Gene ontology analysis

Gene ontology (GO) analysis of genes differentially expressed between clusters, conditions, and cell states, or along trajectory path pseudotimes was carried out using the gProfiler^14^ web tool (https://biit.cs.ut.ee/gprofiler/gost) for all terms against mus musculus or homo sapiens genes (as appropriate) with a term size<1000. The Circos plot was generated using the GOplot package (CRAN). Combined GO analysis was performed using the compareCluster function in clusterProfiler^15^. Statistical significance was evaluated using a 0.05 P-adj threshold.

## Immunofluorescence

Mouse arteries were fixed in 4% formaldehyde (Sigma) for 20 minutes at room temperature (RT), cryopreserved in 30% sucrose in PBS overnight, adjusted for 1 hour in a 50:50 solution of 30% sucrose solution: Optimal Cutting Temperature (OCT) compound (VWR), followed by 1 hour in 100% OCT and snap-frozen in OCT using dry ice. Sections (14 μm for immunostaining, 100 μm for lesional VSMC infiltration analysis) were cut onto Superfrost® Ultra slides (Thermo) and either mounted directly or stained in a humidity chamber protected from light. For immunostaining, cryosections were rinsed in PBS and permeabilised in 0.5% (v/v) Triton X-100 (Sigma Aldrich) for 20 minutes in PBS at room temperature, incubated for 1 hour at RT in blocking buffer [1% (w/v) bovine serum albumin and 10% (v/v) normal goat serum, Dako] before incubation with primary antibodies for NOTCH3 (Abcam, ab23426, 10 μg/mL), VCAM1 (BioLegend, 105702, 10 μg/mL) or isotype control antibodies diluted in blocking buffer overnight at 4°C. Primary antibodies were removed with 3 x 5 minute PBS washes before incubation with Alexa Fluor 647 and Alexa Fluor 750-conjugated secondary antibodies (diluted in blocking buffer) for 1 hour at room temperature. Sections were washed 2 x 5 minutes in PBS, nuclei stained with DAPI (1 μg/mL) for 10 minutes at room temperature, before rinsing in PBS and mounting in RapiClear 1.52 (Sunjin Lab).

## Confocal imaging

Confocal imaging was performed using an SP8 (VSMC plaque infiltration analysis) or Stellaris (immunostaining analysis) laser scanning microscope (Leica) with a 20x lens in sequential, resonant, tile scan mode with 3 μm distance between Z-stack sections. Laser lines and detector settings were selected to avoid spectral overlap; VSMC infiltration analysis (SP8): sequence 1 (405/410-462, DAPI), sequence 2 (458/462-483, CFP and 555/565-632, RFP), sequence 3 (514/520-558, YFP), and sequence 4 (488/490-507, GFP); seven-colour immunostaining (Stellaris): sequence 1 (405/410-440, DAPI and 638/643-710, Alexa Fluor 647), sequence 2 (448/453-475, CFP and 730/735-850, Alexa Fluor 750), sequence 3 (488/493-509, GFP), sequence 4 (514/519-540, YFP) and sequence 5 (561/566-610, RFP). Data was acquired at an optical section resolution of 1024 x 1024 and tiles were stitched using the mosaic merge function in LASX software (Leica). Image analysis was done using Imaris software (9.1.2) to adjust brightness and contrast; isotype control and primary antibody-stained sections were subject to identical manipulations.

## Scoring of lineage-traced VSMC sections

Quantification of VSMC infiltration in plaques was done using 100 μm plaque sections analysed previously to assess VSMC clonality^2^. A total of 155 unique plaques from the ascending and descending aorta, aortic arch and carotid arteries of 7 animals (4 at week 6.5 and 3 at week 11) were imaged (Supplementary Table 2). Plaques with suboptimal clearing (5) were excluded from the analysis, hence a total of 150 were included in the analysis. Lesions were qualitatively classified into size groups based on extent of lumen occlusion, and cap presence scored when contiguously arranged cells with elongated nuclei were present at the luminal edge. Elastic lamina breaks were scored in lesions where image quality was sufficient to detect localised absence of autofluorescence from the internal elastic lamina (IEL). Plaque annotation was done based on DAPI signals only, and VSMC investment was subsequently scored based on Confetti signals. Quantification of VCAM1 and NOTCH3 staining in Confetti+ cells in plaques was done after immunostaining of 14 μm sections from a subset of plaques (n=16, total 26 clones) from animals after 11 weeks HFD (n=3), see Supplementary Table 2. VSMC location was scored as "luminal edge" when present within three cell-widths of the endothelial boundary or if elongated and part of a luminally located clone, "internal elastic lamina (IEL) adjacent (IEL-A)” when inside the plaque but in direct contact with the IEL, and "core" if meeting neither of these criteria. Quantification of VCAM1 and NOTCH3 staining in Confetti+ cells after injury was performed in 3 control vessels or diseased regions of vessels from 3 injured animals per timepoint. Expression of VCAM1 and/or NOTCH3 by individual VSMCs was confirmed by navigating through multiple *Z-*planes.

## Human tissue

Anonymised human artery samples were obtained from patients undergoing carotid endarterectomy, cardiac transplant, valve replacement or by donation after circulatory death. All human tissue acquisition was done under informed consent using protocols approved by the Cambridge or Huntingdon Research Ethical Committee.

## Spatial transcriptomics

A plaque-containing aortic sample from a 54 year old male organ donor was snap-frozen by fully submerging in an isopentane bath cooled by dry ice until frozen, embedded in OCT (2-8°C), sectioned and four consecutive sections analysed in parallel using VISIUM technology (cat 1000184) according to manufacturer's instructions (10X). Sequencing data was aligned to a 10X-supplied human reference genome (GRCh38-3.0.0) using Space Ranger (v.1.0). Downstream analysis was performed with R (v.4.0.3). The Spaniel R package^16^ (v.1.2.0) was used to import the data into SingleCellExperiment objects and was also used to create spot images. Any spots with fewer than 200 UMIs were removed. The SingleCellExperiment objects were then converted into SeuratObjects for downstream analysis using Seurat (v.3.1.3). Individual replicates were normalised using the NormaliseData function followed by the identification of the top 200 highly variable genes using the FindVariableFeatures function. Replicates (from consecutive sections) were integrated using the FindIntegrationAnchors method, with dims = 1:20 and k.filter = 50. Following this, the data was processed with the following functions: data was scaled with ScaleData, RunPCA was used with npcs = 30, RunUMAP with dims = 1:15 and FindNeighbors with k.param = 10. The FindClusters function was used to cluster spots with a resolution of 0.4. The Seurat function FindAllMarkers was used to identify marker genes for each cluster using default settings (log_2_FC>0.5). All parameters were selected as default unless otherwise stated.

## Immunohistochemistry

Human arteries were formaldehyde-fixed and paraffin-embedded (FFPE) and sections (4 μm) were dewaxed, processed for antigen retrieval and stained as described^2^. Sections were co-stained for αSMA (DAKO, M0851, 1:400), detected with biotin-coupled anti-Mouse (DAKO, E0433) using Vectastain avidin-coupled alkaline phosphatase with Blue AP substrate solution (Vector Labs), and PAR1 (Novus Biologicals, N2-11, 1:200), that was detected with HRP-conjugated anti-Rabbit (Cell Signaling Technology, 8114, using 3,3'-diaminobenzidine tetrahydrochloride (DAB) as peroxidase substrate, SignalStain).

## Cell culture

Human VSMC (hVSMC) cultures were generated from aortas of patients undergoing cardiac transplant or aortic valve replacement. After manually removing the endothelial layer and adventitia, the medial layer was cut into 2-3 mm² pieces, placed into 6-well plates containing 1 ml media [DMEM supplemented with 20% fetal calf serum (FCS),100 U/mL penicillin, 100 µg/mL streptomycin] and cultured to allow cells to migrate out of the tissue (1-2 weeks). After establishment, cells were cultured in hVSMC-specific medium (Promocell, SMC-GM2) supplemented with 100U/mL penicillin, 100µg/mL streptomycin and were studied at passages 2–10. Treatment with thrombin (0.5, 1 or 2 U/ml) and Factor Xa (2.5 μg/ml) was performed in serum-free media. To transiently silence PAR1, hVSMCs were transfected with 50 nM human PAR1-specific siRNA (ON-TARGETplus® SMART Pool, Dharmacon, L-005094-00-0005) or non-targeting control siRNA (ON-TARGETplus® Control Pool, nontargeting pool, Dharmacon, D-001810-10-05) for 72 hours using Lipofectamine RNAiMAX transfection reagent (Invitrogen). Cells were then washed prior to 24 hour thrombin treatments, followed by downstream analysis.

## Bulk expression analysis

Total RNA was isolated using the RNeasy Mini kit (Qiagen), reverse transcribed using QuantiTect Reverse Transcriptase (Qiagen), and cDNA corresponding to 10 ng RNA was analysed using quantitative, real-time, reverse transcription PCR (RT-qPCR) with SsoAdvanced Universal SYBR Green Supermix (Biorad, primer sequences listed in Supplementary Table 2). HMBS was used as a housekeeping gene for data normalisation. Bulk RNA-seq was conducted with RNA isolated from 3 different female hVSMC isolates following a 24 hour thrombin treatment in serum-free medium. Libraries were prepared from oligo-dT-purified mRNA and sequenced on an Illumina Novaseq 6000 (150 bp paired end reads). Raw data reads were trimmed with Trim Galore (v.0.6.7), and aligned to the human genome (GRCh38) with Kallisto (v.0.46.2). Trimmed mean of M-values (TMM) normalization was then conducted. Differential expression analysis was performed using the voom normalisation workflow of the limma package^17^ (Bioconductor) with cell line donor as a covariate. Linear models were fitted to the normalised data, and empirical Bayes moderation was applied to the standard errors of the estimated log-fold changes. Contrast matrices were specified to identify differentially expressed genes between treatment groups. An FDR-corrected P-adj<0.05 was considered statistically significant. The thrombin-upregulated gene module for expression analysis in the scRNA-seq data was generated by converting human gene orthologues to mouse using the gProfiler web tool and using these orthologues with the AddModuleScore function in Seurat.

To analyse PAR1 protein expression, whole cell protein lysates were prepared in RIPA buffer freshly supplemented with proteinase inhibitors (Millipore) and phosphatase inhibitors (Millipore). Protein concentration was determined using the BCA method (23227, Pierce BCA protein assay kit, Thermo Fisher). Immunoblotting was performed according to standard conditions, using gradient (4-12%) polyacrylamide gels, methanol-based wet transfer and chemiluminescence detection (Amersham ECL detection reagent, GE Healthcare). Primary antibodies for PAR1 (Santa Cruz, ATAP2, 1:2000), and GAPDH (loading control, Cell Signaling Technologies, 2118, 1:4000) were detected using HRP-labelled secondary antibodies: goat-anti-rabbit (7074S, Cell Signaling Technology).

## Data availability

The bulk (GSE274393), spatial (GSE274572) and single cell RNAseq datasets (GSE274572) generated in this study have been deposited to the Gene Expression Omnibus (GEO) repository. The scRNA-seq datasets from VSMC-lineage-labelled plaque cells from HFD-fed animals are available from GEO (GSE155513). Source data is available upon request.

## Statistical analysis

Statistical analysis was performed in *R* or *GraphPad Prism*. Statistically significance of differences was assessed using Chi-squared analysis for categorical variables, and the analysis was done using raw count values. Kruskal-Wallis testing followed by Mann-Whitney testing for pairwise comparison, with Bonferroni multiple testing correction, was done for continuous variables that did not pass normality (Shapiro–Wilk) and equal variance (Bartlett or Levine) tests. Group sizes represent biologically independent samples (different donors, different mice, plaques or clones as specified), rather than technical replicates. An adjusted P-value (P-adj) less than 0.05 was considered statistically significant. Details on statistical testing for single cell and bulk RNA-seq dataset analysis is provided above.

## Supplementary References

1 Chappell, J., Harman, J. L., Narasimhan, V. M., Yu, H., Foote, K., Simons, B. D., Bennett, M. R. & Jørgensen, H. F. *Circ Res* **119**, 1313-1323, doi:10.1161/circresaha.116.309799 (2016).

2 Worssam, M. D., Lambert, J., Oc, S., Taylor, J. C. K., Taylor, A. L., Dobnikar, L., Chappell, J., Harman, J. L., Figg, N. L., Finigan, A., Foote, K., Uryga, A. K., Bennett, M. R., Spivakov, M. & Jørgensen, H. F. *Cardiovasc Res* **119**, 1279-1294, doi:10.1093/cvr/cvac138 (2023).

3 Pan, H., Xue, C., Auerbach, B. J., Fan, J., Bashore, A. C., Cui, J., Yang, D. Y., Trignano, S. B., Liu, W., Shi, J., Ihuegbu, C. O., Bush, E. C., Worley, J., Vlahos, L., Laise, P., Solomon, R. A., Connolly, E. S., Califano, A., Sims, P. A., Zhang, H., Li, M. & Reilly, M. P. *Circulation* **142**, 2060-2075, doi:10.1161/CIRCULATIONAHA.120.048378 (2020).

4 Hafemeister, C. & Satija, R. *Genom Biol* **20**, 296, doi:10.1186/s13059-019-1874-1 (2019).

5 Satija, R., Farrell, J. A., Gennert, D., Schier, A. F. & Regev, A. *Nature Biotechnology* **33**, 495-502, doi:10.1038/nbt.3192 (2015).

6 Ovchinnikova, S. & Anders, S. *Genome Res* **30**, 749-756, doi:10.1101/gr.251447.119 (2020).

7 Street, K., Risso, D., Fletcher, R. B., Das, D., Ngai, J., Yosef, N., Purdom, E. & Dudoit, S. *BMC Genomics* **19**, 477, doi:10.1186/s12864-018-4772-0 (2018).

8 Lun, A. & D., R. (Bioconductor, <https://bioconductor.org/packages/release/bioc/html/SingleCellExperiment.html>, 2020).

9 Hastie, T. (CRAN, <https://cran.r-project.org/web/packages/gam/index.html>, 2019).

10 Kolde, R. (CRAN, <https://cran.r-project.org/web/packages/pheatmap/index.html>, 2019).

11 Wolf, F. A., Hamey, F. K., Plass, M., Solana, J., Dahlin, J. S., Göttgens, B., Rajewsky, N., Simon, L. & Theis, F. J. *Genom Biol* **20**, 59, doi:10.1186/s13059-019-1663-x (2019).

12 Wolf, F. A., Angerer, P. & Theis, F. J. *Genom Biol* **19**, 15, doi:10.1186/s13059-017-1382-0 (2018).

13 Philippe, A. (Pypi, <https://pypi.org/project/anndata2ri/#description>, 2020).

14 Raudvere, U., Kolberg, L., Kuzmin, I., Arak, T., Adler, P., Peterson, H. & Vilo, J. *Nucleic Acid Res* **47**, W191-W198, doi:10.1093/nar/gkz369 (2019).

15 Yu, G., Wang, L. G., Han, Y. & He, Q. Y. *Omics* **16**, 284-287, doi:10.1089/omi.2011.0118 (2012).

16 Queen, R., Crosier, M., Eley, L., Kerwin, J., Turner, J. E., Yu, J., Alqahtani, A., Dhanaseelan, T., Overman, L., Soetjoadi, H., Baldock, R., Coxhead, J., Boczonadi, V., Laude, A., Cockell, S. J., Kane, M. A., Lisgo, S. & Henderson, D. J. *PLoS Genet* **19**, e1010777, doi:10.1371/journal.pgen.1010777 (2023).

17 Ritchie, M. E., Phipson, B., Wu, D., Hu, Y., Law, C. W., Shi, W. & Smyth, G. K. *Nucleic Acids Res* **43**, e47, doi:10.1093/nar/gkv007 (2015).
